# Accuracy of a Recent Regularized Nuclear Potential

**DOI:** 10.1021/acs.jctc.3c00530

**Published:** 2023-06-24

**Authors:** Susi Lehtola

**Affiliations:** † Department of Chemistry, University of Helsinki, P.O. Box 55, FI-00014 Helsinki, Finland

## Abstract

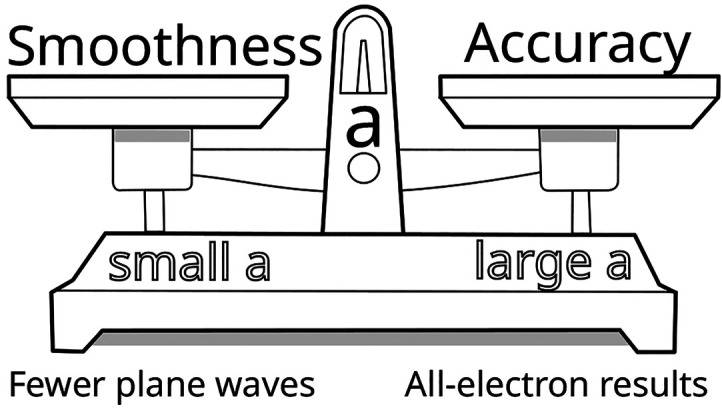

F. Gygi recently suggested an analytic, norm-conserving, regularized nuclear potential to enable all-electron plane-wave calculations [Gygi J. Chem. Theory Comput.2023, 19, 1300–1309.]3675729110.1021/acs.jctc.2c01191PMC9979607. This potential *V*(*r*) is determined by inverting the Schrödinger equation for the wave function Ansatz ϕ(***r***) = exp[−*h*(***r***)]/√π with *h*(***r***) = *r* erf(*ar*) + *b* exp(−*a*^2^*r*^2^), where *a* and *b* are parameters. Gygi fixes *b* by demanding ϕ to be normalized, with the value *b*(*a*) depending on the strength of the regularization controlled by *a*. We begin this work by re-examining the determination of *b*(*a*) and find that the original 10-decimal tabulations of Gygi are only correct to 5 decimals, leading to normalization errors in the order of 10^–10^. In contrast, we show that a simple 100-point radial quadrature scheme not only ensures at least 10 correct decimals of *b* but also leads to machine-precision level satisfaction of the normalization condition. Moreover, we extend Gygi’s plane-wave study by examining the accuracy of *V*(*r*) with high-precision finite element calculations with Hartree–Fock and LDA, GGA, and meta-GGA functionals on first- to fifth-period atoms. We find that although the convergence of the total energy appears slow in the regularization parameter *a*, orbital energies and shapes are indeed reproduced accurately by the regularized potential even with relatively small values of *a*, as compared to results obtained with a point nucleus. The accuracy of the potential is furthermore studied with *s*-*d* excitation energies of Sc–Cu as well as ionization potentials of He–Kr, which are found to converge to sub-meV precision with *a* = 4. The findings of this work are in full support of Gygi’s contribution, indicating that all-electron plane-wave calculations can be accurately performed with the regularized nuclear potential.

## 1 Introduction

Solid-state systems are traditionally modeled with density functional theory^1,2^ (DFT) with plane-wave basis sets of the form χ_***G***_(***r***) = Ω^–1/2^*e*^*i****G***·***r***^, where ***G*** is a reciprocal lattice vector and Ω is the volume of the simulation box.^3^ Importantly, plane-waves form a systematically improvable basis set, whose accuracy is determined by a single parameter: the plane-wave kinetic energy cutoff *E*_cut_. The basis set of plane-waves ***G*** corresponding to a given cutoff is concisely defined by , and the complete basis set limit can in principle be reached by converging the calculation with respect to *E*_cut_.

However, the plane-wave basis set has a fixed resolution. This is an issue, since the resolution is the same close to nuclei, where the electronic wave function undergoes rapid oscillations and where thereby an extremely fine spatial resolution is needed, as in empty regions of space where the wave function is typically smooth, lacking high-frequency components. An accurate description of the core region requires extremely large values of *E*_cut_, resulting in prohibitive numbers of plane-waves that render calculations untractable.

Plane-wave methods traditionally address this problem by removing the need to describe the rapid oscillations near the nuclei by employing various forms of pseudopotentials,^4−10^ a term that we use here in the broadest sense that also includes the projector-augmented wave (PAW) method.^11^ These pseudopotentials lead to smooth pseudowave functions, which can be accurately computed with moderate values of *E*_cut_, thereby enabling powerful applications of DFT to the study of solid-state systems.^12^

However, introducing the pseudopotential introduces an approximation, which may not always be accurate. For instance, it is common practice to employ pseudopotentials determined for generalized gradient approximation (GGA) functionals also in calculations using meta-GGA functionals, even though GGA and meta-GGA functionals do not reproduce the same core orbitals. The self-consistent use of meta-GGA functionals with pseudopotentials is an active area of study,^13−17^ and fully self-consistent methods for meta-GGA functionals may become widely available in the future.

Another option for achieving full self-consistency is to avoid the need for pseudopotentials altogether. For instance, real-space methods allow employing different levels of resolution in different regions of space, allowing the use of denser basis sets close to nuclei and making all-electron calculations tractable.^18^ It was also recently pointed out that all-electron calculations could be made tractable with plane-waves by eliminating the nuclear cusp, which is hard to describe with plane-waves, by suitable modifications to the nuclear Coulomb potential.

In ref (19), Gygi looked for such a smooth analytic nuclear potential that would be amenable for all-electron calculations with plane-waves. To guarantee its accuracy, this potential should yield the exact eigenvalue *E* = −1/2 for the hydrogenic Schrödinger equation
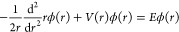
1while requiring differentiability of ϕ(*r*) at *r* = 0 and the correct asymptotic limit ϕ(*r*) → exp(−*r*)/√π for *r* → ∞. Gygi’s solution inverts *V*(*r*) from eq 1 using the Ansatz for the 1*s* orbital
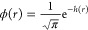
2where *h*(*r*) is unknown. Gygi finds that the function
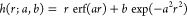
3satisfies the requirements posed above and the arising regularized potential to be given by
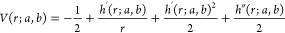
4

Equation 3 has two parameters: *a* and *b*. Gygi fixes the *b* parameter by following Hamann et al.^4^ and requiring ϕ(*r*) to be normalized
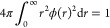
5Scaling with the nuclear charge *Z* lead Gygi to postulate that the potential for *Z* > 1 is given by
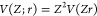
6

Gygi computed atomic energies for the H and Be atoms in ref (19) within the local density approximation (LDA) and found them to be in *μE*_*h*_ level agreement with the values of Kotochigova et al.^20,21^ The study then proceeded to plane-wave calculations on various polyatomic systems—diamond, silicon, MgO, solid argon, and liquid water—where the convergence of orbital energies, band gaps, ionic forces, and stress tensors was studied.

In this contribution, we examine Gygi’s regularized potential using high-precision atomic calculations including all electrons. In addition to the LDA, we also consider Hartree–Fock (HF), generalized gradient approximation (GGA), and meta-GGA level density functional approximations of total energies.

The layout of this work is as follows. We begin in section 2 by describing the implementation of the regularized potential in the HelFEM program,^17,18,22−24^ which enables all-electron finite element approaches that routinely afford sub-*μE*_*h*_ accuracy in total energies for atoms for a variety of functionals and offers a good starting point for studying the accuracy of Gygi’s regularized potential, as well. Next, in section 3, we study the accuracy of total energies as well as orbital energies and shapes. The computational details are outlined in section 3.1, the accuracy of total energies is studied in section 3.2, and the examination of the accuracy of orbital energies and shapes is carried out in section 3.3. These calculations are carried out on the He, Be, Ne, Mg, Ar, Ca, Zn, Kr, Sr, Cd, and Xe atoms, which suffice to study the essential features of the regularized potential. These results are extended with studies of relative energies in section 4: *s*-*d* excitation energies of first-row transition metal atoms are studied in section 4.1, and ionization potentials for He–Kr are studied in section 4.2. The study concludes in a short summary and conclusions in section 5.

## 2 Implementation

We have implemented the potential defined by eqs 3, 4, and 6, in HelFEM. We determine *b*(*a*) from eq 5 using the bisection method and radial quadrature with *N* = 100 points with the default scheme of ref (25), which is given by the M3 grid of Treutler and Ahlrichs^26^ without atomic size adjustment (ξ = 1) combined with the Gauss–Chebyshev quadrature formulas of the second kind of Pérez-Jordá et al.^27^ that have simple closed-form expressions, see eqs (31)–(33) in ref (27).

Gygi tabulated *b*(*a*) with 10 decimals in ref (19). The values *b*(*a*) from our implementation are compared with Gygi’s in Table 1. Because of the notable discrepancies observed in the values of *b*(*a*)—up to half the decimals disagree—we carried out arbitrary precision calculations in Maple 2020. We found that employing 20 digit precision in Maple yielded *b* converged to 10 decimals. We observe that our simple scheme yields values for *b* that are in full agreement with those from Maple, while the tabulation of Gygi—whose provenance is not described—is not converged to the number of decimals (10) given in ref (19), several values only being accurate to five decimals.

**Table 1:** Comparison of *b* Values from the Quadrature Implementation Used in the Present Work vs the Values Given by Gygi in Ref (19)^a^

*a*	*b*(*a*), PW	*b*(*a*) from ref (19)	*b*(*a*), Maple 2020
1	**3.6442293860**e-01	**3.64422938**56e-01	3.6442293860e-01
2	**1.9653418941**e-01	**1.96534189**82e-01	1.9653418941e-01
3	**1.3433604767**e-01	**1.34336047**53e-01	1.3433604767e-01
4	**1.0200558632**e-01	**1.0200558**466e-01	1.0200558632e-01
5	**8.2208090847**e-02	**8.220809**1118e-02	8.2208090847e-02
6	**6.8842562733**e-02	**6.88425**55167e-02	6.8842562733e-02
7	**5.9213661071**e-02	**5.92136**52850e-02	5.9213661071e-02
8	**5.1947028410**e-02	**5.1947028**250e-02	5.1947028410e-02
9	**4.6268541343**e-02	**4.62685**59218e-02	4.6268541343e-02
10	**4.1708946804**e-02	**4.17089**13494e-02	4.1708946804e-02
11	**3.7967255428**e-02	**3.79672**27308e-02	3.7967255428e-02
12	**3.4841536898**e-02	**3.48415**73775e-02	3.4841536898e-02

a For comparison, *b* values solved with guaranteed precision with Maple 2020 (present work, PW) are also shown; digits of the two implementations that coincide with the Maple reference value are shown in bold.

To assess the practical importance of the errors in the *b* values used in ref (19), we have computed the errors in the normalization arising from the various *b* values of Table 1 with Maple; these results are shown in Table 2. The errors in the normalization of the Ansatz of eq 2 are smaller than 10^–10^ also with Gygi’s approximate values for *b*, indicating that the values reported in ref (19) are likely sufficiently accurate not to cause severe issues in the validity of the results.

**Table 2:** Comparison of Errors in Normalization *ΔN* = 4π∫_0_^∞^*r*^2^ϕ(*r*)^2^d*r* – 1 of ϕ(*r*) of Eq 2 with the Values *b* of Table 1 of the Present Work (PW) and the Values of Gygi in Ref (19), Evaluated with Maple 2020 with 25 Digits^a^

*a*	PW	ref^19^	PW, fp
1	1.676e-13	2.118e-11	6.075e-17
2	7.479e-13	–6.321e-11	2.601e-16
3	–1.786e-13	8.788e-12	1.175e-16
4	1.368e-13	5.335e-11	5.450e-17
5	–8.680e-15	–4.950e-12	–1.042e-16
6	5.135e-15	8.574e-11	1.495e-16
7	–2.430e-15	6.176e-11	9.851e-17
8	–1.722e-15	8.355e-13	2.504e-16
9	–1.731e-15	–6.773e-11	2.306e-16
10	1.136e-15	9.429e-11	2.547e-16
11	–4.674e-16	6.101e-11	3.285e-16
12	3.256e-16	–6.268e-11	2.372e-16

a For comparison, the last column shows the values obtained using the full precision (fp) value of *b* with 15 decimals, similar to what is used internally in HelFEM.

In contrast, if one employs values of *b* that are really correct to 10 decimal places, the normalization errors are reduced by a few orders of magnitude. However, the implementation in HelFEM does not truncate *b* to 10 decimal places but instead determines *b* to near machine precision. Inserting the value of *b* printed out by HelFEM with 15 decimals to Maple shows that ϕ(*r*) is practically normalized to within machine precision, the largest absolute value in the rightmost column of Table 2 being 1.5 times machine epsilon ϵ ≈ 2.2 × 10^–16^. We therefore can conclude that our simple scheme to automatically determine *b*(*a*) is sufficient to achieve machine precision and that pretabulation of *b*(*a*) is thereby not necessary.

## 3 Accuracy of Total and Orbital Energies and Shapes

### 3.1 Computational Details

Employing the above numerical scheme for finding *b*(*a*) in an automated fashion, we have calculated nonrelativistic total energies for HF, the Perdew–Wang 1992 LDA (PW92),^28−30^ the Perdew–Burke–Ernzerhof (PBE) GGA,^31,32^ as well as the TASKCC meta-GGA functional^33,34^ recommended by Lebeda et al.^35^ with the normal Coulomb potential of a point nucleus *E*^point^ as well as the regularized potential of eq 4 (*E*^regularized^(*a*)) with various values for the regularization parameter *a*. All density functionals are evaluated in HelFEM with Libxc.^36^

We find that the calculations employing the regularized potential converge more slowly to the complete basis set (CBS) limit than the calculations with the point nucleus, when the default radial grid optimized for point nuclei is used. This means that more radial finite element basis functions are required to reach the CBS limit in calculations employing the regularized potential.

Following the grid analyses performed in refs (22) and (24), we considered reoptimizing the “exponential” finite element grid^22^
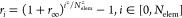
7where *r*_∞_ = 40*a*_0_ is the employed value for the practical infinity beyond which all wave functions vanish and *N*_elem_ is the used number of elements, by retuning the *z* parameter that controls the composition of the grid from the default value *z* = 2 optimized for the point nucleus.^22,24^ We found that calculations with the regularized potential favor denser grids close to the nucleus than those employing a point nucleus, that is, large values of *z* (not shown). We attribute the increased sensitivity in the region close to the nucleus to the more complicated form of eq 4 over the *r*^–1^ Coulomb interaction. However, grids with *z* ≫ 2 tend to lead to poorly convergent self-consistent field calculations, and we choose to employ the default value *z* = 2 also in the present calculations.

We found that when employing a 10-node Hermite interpolating polynomial basis,^24^ which corresponds to employing a 19th order polynomial scheme, all calculations are converged to the CBS limit—which we define as 0.1 *μE*_*h*_ accuracy—when 25 radial elements are employed. For all the systems studied in this section, calculations with 30 radial elements yield the same total energy to 7 decimals. We also observe that the regularized potential results in a lack of a nuclear cusp in the wave function.

### 3.2 Accuracy of Total Energies

We will next proceed to discuss the errors in total energies caused by the regularized potential approximation. We define this regularization error by
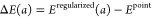
8and use it to assess the convergence of the total energy to the point nucleus value. Plots of *ΔE*(*a*) for all studied atoms and functionals are available in the Supporting Information; we will only present some of the figures in the main text to exemplify our findings.

**Figure 1:**
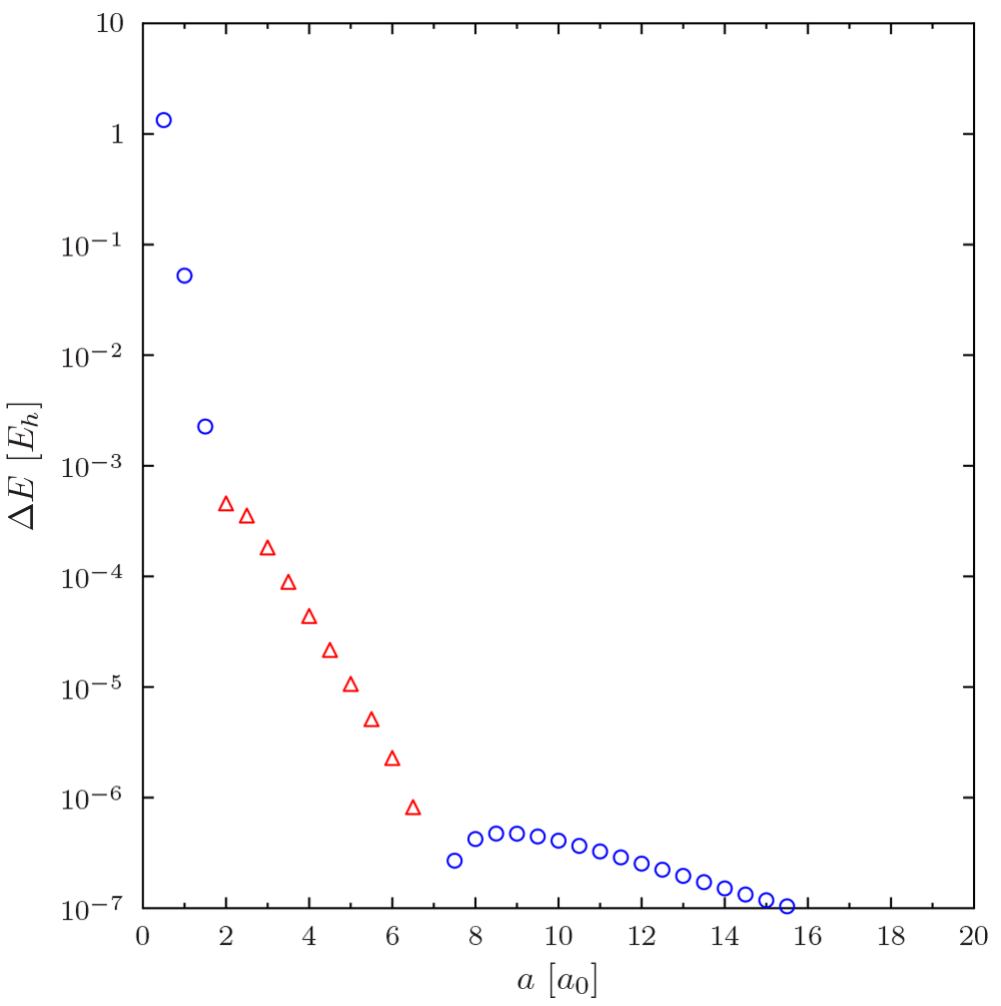
Regularization error in the HF total energy of Ne. Note the use of a logarithmic *y* axis. Positive energy errors (*E*^regularized^ > *E*^Coulomb^) are shown with blue squares, and negative energy errors (*E*^regularized^ < *E*^Coulomb^) are shown with red triangles.

Depending on the functional, the error in the total energy may be positive or negative, as is demonstrated by the HF calculation on Ne in Figure 1. The data in the figure show that there are sharp minima in *ΔE*(*a*), possibly caused by fortuitous error cancellation when the structure of the regularized potential matches the shell structure of the atom. These artifactual error minima may complicate convergence studies with the regularized potential, but these issues appear to only affect the lighter atoms. We observe that total energies can be reproduced to *μE*_*h*_ accuracy when a sufficiently large value for *a* is employed.

**Figure 2:**
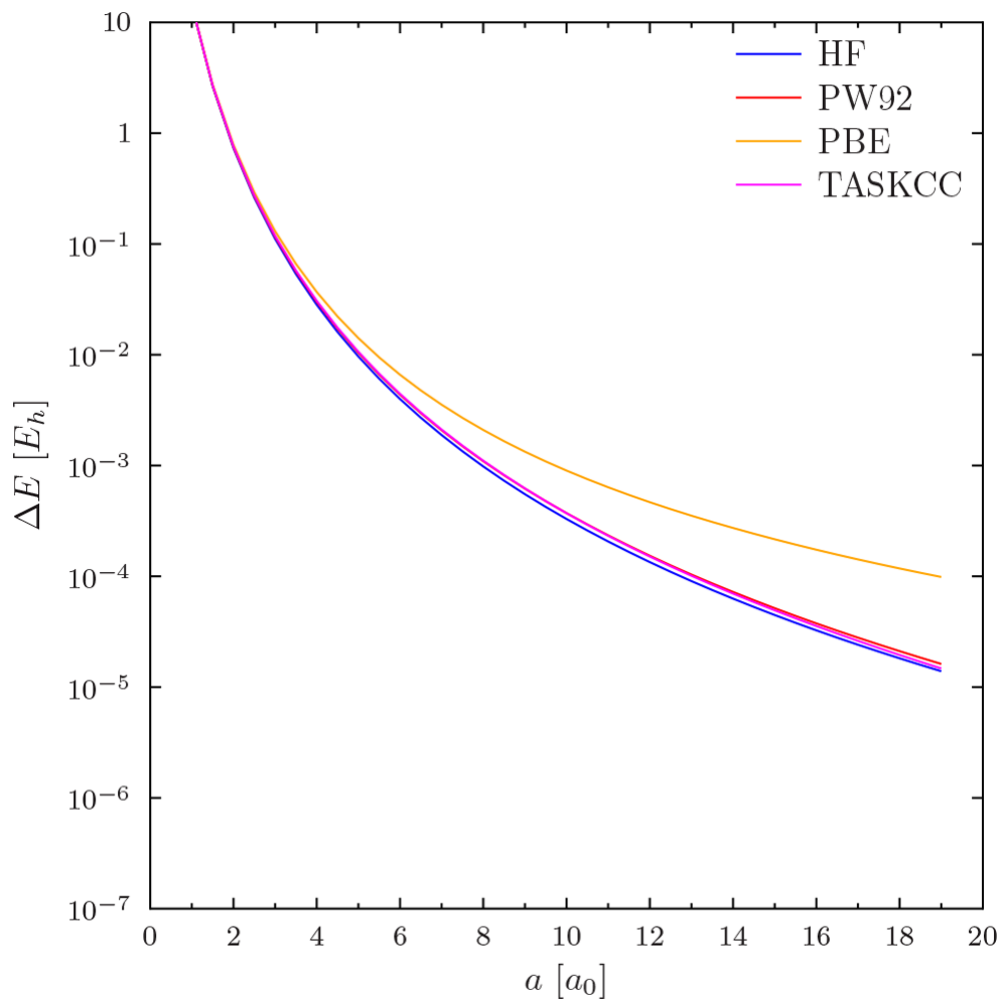
Regularization errors in the HF, PW92, PBE, and TASKCC total energies of Xe. Note the use of a logarithmic *y* axis. All energy differences are positive.

Heavier atoms appear to lead to larger differences in total energy. The differences in total energy *ΔE*(*a*) are positive for all studied values of *a* ∈ [0.5, 19] from Mg onward, and the convergence plots appear similar for all atoms and functionals. However, the level of convergence in the total energy depends on the functional. This is exemplified by the HF, PW92, PBE, and TASKCC calculations on Xe in Figure 2, which has the typical convex-type form of most of our results.

We note that the convergence to the CBS limit is slow in *a*. The data in Figure 2 show that the error decays more slowly with PBE than with the other studied functionals. The total energy is converged to 0.1 m*E*_*h*_ level accuracy for PBE with the largest value of *a* considered in this study (*a* = 19), while the differences for the other functionals are in the tens of microhartrees. For comparison, ref (19) employed *a* = 3 or *a* = 4 for non-hydrogen atoms and up to *a* = 8 for hydrogen in polyatomic calculations.

### 3.3 Accuracy of Orbital Energies and Shapes

Although total energies converge slowly, we do find that orbital energies and orbital shapes are indeed accurately captured by the regularized approximation; tables of orbital energies for all atoms and functionals are available in the Supporting Information. For example, the errors in orbital energies of Xe with the TASKCC functional are shown in Table 3 for various values of *a*. Even small values of *a* that correspond to *E*_*h*_ level errors in the total energy as seen from Figure 2 afford accurate orbital energies. For instance, while *a* = 2 reproduces a total energy that differs by 0.76*E*_*h*_ from the point nucleus value, the differences in orbital energies are an order of magnitude smaller.

**Table 3:** Errors in Orbital Energies in *E*_*h*_ for the Xe Atom Computed with TASKCC and the Regularized Potential with Various Values of *a*^a^

Energy	*a* = 1.0	*a* = 2.0	*a* = 3.0	*a* = 5.0	*a* = 7.0	point nucleus
1*s*	–1.3075	–0.0864	–0.0126	–0.0008	–0.0001	–1212.0214
2*s*	1.3285	0.0370	0.0035	0.0001	0.0000	–184.6493
2*p*	1.1213	0.0662	0.0107	0.0010	0.0002	–173.6861
3*s*	0.3128	0.0090	0.0008	0.0000	0.0000	–37.9716
3*p*	0.1536	0.0118	0.0020	0.0002	0.0000	–33.3301
3*d*	–0.0712	–0.0046	–0.0007	–0.0001	–0.0000	–24.6824
4*s*	0.0637	0.0017	0.0001	0.0000	–0.0000	–6.9179
4*p*	0.0217	0.0019	0.0003	0.0000	0.0000	–5.2477
4*d*	–0.0178	–0.0011	–0.0002	–0.0000	–0.0000	–2.3639
5*s*	0.0066	0.0001	–0.0000	–0.0000	–0.0000	–0.7190
5*p*	–0.0000	0.0000	0.0000	0.0000	0.0000	–0.3306
*ΔE*	14.4457645	0.7612456	0.1184265	0.0106325	0.0021111	–7233.3416395

a The values obtained with the Coulomb potential of the point nucleus are shown in the last column. For comparison, the last row shows the errors in total energy *ΔE* from the point nucleus value shown in the last column.

We also find that the shapes of the orbitals are reproduced accurately already with modest values of *a*. The positions of the orbital density maxima, defined for radial orbital ψ_*i*_(*r*) as
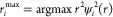
9are shown in Table 4 for Xe with the TASKCC functional; the results for all atoms and functionals are available in the Supporting Information. Similarly to the orbital energies discussed above, the positions of the orbital density maxima are already correct to millibohr with *a* = 2. Similar findings also apply to the radial moments of the orbitals ⟨*r*^*n*^⟩ for *n* ∈ [−2, – 1, 1, 2, 3] (not shown).

**Table 4:** Errors in Positions of Orbital Density Maxima in bohr for the Xe Atom Computed with TASKCC and the Regularized Potential with Various Values of *a*^a^

Energy	*a* = 1.0	*a* = 2.0	*a* = 3.0	*a* = 5.0	*a* = 7.0	point nucleus
1*s*	0.000115	–0.000150	–0.000002	–0.000000	0.000000	0.018648
2*s*	0.000638	0.000020	0.000002	0.000000	0.000000	0.102941
2*p*	0.000634	0.000037	0.000006	0.000001	0.000000	0.080418
3*s*	0.001579	0.000050	0.000005	0.000000	0.000000	0.292393
3*p*	0.000996	0.000071	0.000012	0.000001	0.000000	0.278905
3*d*	–0.000118	–0.000011	–0.000002	0.000000	0.000000	0.226757
4*s*	0.003284	0.000102	0.000010	0.000000	0.000000	0.689832
4*p*	0.001849	0.000145	0.000025	0.000002	0.000001	0.706492
4*d*	–0.000837	–0.000059	–0.000009	–0.000001	–0.000000	0.746263
5*s*	0.008732	0.000256	0.000024	0.000001	0.000000	1.709528
5*p*	0.004292	0.000377	0.000067	0.000007	0.000002	1.937097

a The values obtained with the Coulomb potential of the point nucleus are shown in the last column.

## 4 Accuracy of Relative Energies

### 4.1 Accuracy of Excitation Energies

Having established the fast convergence of orbital expectation values with respect to *a*, one might ask whether the same also holds for relative energies. In addition to being a stringent check for the accuracy of density functionals,^37−39^ the *s*-*d* excitation energies of first-row transition metals (*s*^2^*d*^*n*–1^ → *s*^1^*d*^*n*^) are often used to check the reliability of basis sets^40,41^ and pseudopotentials, as they are directly related to the complex chemistry of transition metals. We note that transition metal systems were not studied in ref (19).

Employing spherically symmetric densities for the atoms Sc–Cu in a spin-unrestricted formulation with 25 radial elements as in section 3,^17,23^ we determine the accuracy of the excitation energies
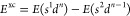
10by computing their differences from the corresponding excitation energies for a point nucleus
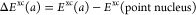
11For reference, approximate point nucleus values are given in Table 5. We find not only that the *s*^2^*d*^*n*–1^ and the *s*^1^*d*^*n*^ states flip order for small values of *a* for many atoms, but also that the order is correctly reproduced when a sufficiently large value of *a* is used (not shown).

**Table 5:** *s*-*d* Excitation Energies in eV for Point Nuclei from Spin-Unrestricted Calculations Employing Spherical Densities

	Sc	Ti	V	Cr	Mn	Fe	Co	Ni	Cu
PW92	0.66	–0.30	–1.20	–2.05	1.04	0.16	0.71	–1.10	2.40
PBE	0.65	–0.35	–1.28	–2.17	1.12	0.23	0.65	–1.18	2.38
TASKCC	0.84	–0.40	–1.56	–2.64	2.03	1.07	0.17	1.39	2.59
r^2^SCAN	0.52	–0.60	–1.66	–2.67	1.87	0.76	0.38	–1.27	2.59

**Figure 3:**
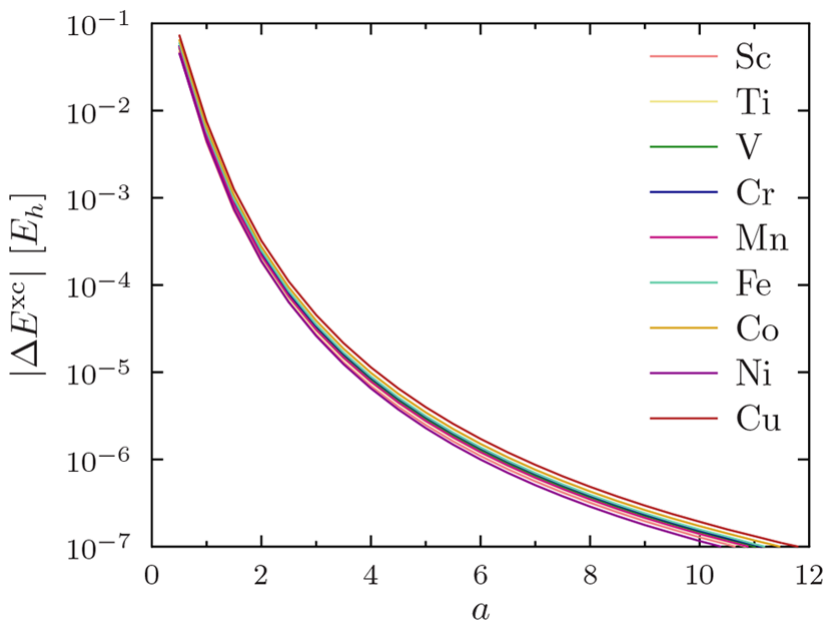
Convergence of *s*-*d* excitation energies *E*^xc^ with increasing regularization parameter *a*. Results are shown for the PBE functional; other functionals yield analogous results. Note the use of a logarithmic *y* axis.

We furthermore observe that *ΔE*^xc^ often has the same sign for the studied range of *a*, implying monotonic convergence of the excitation energy, and that some exceptions also exist where the sign of the error changes at a small value of *a* (not shown). For this reason, it suffices to demonstrate the rapid convergence of |*ΔE*^xc^|, shown in Figure 3 for the PBE functional, as PW92, TASKCC, and r^2^SCAN were found to yield similar results (not shown).

As expected, the data for all atoms Sc–Cu appear similar. At small *a*, the potential for erroneous state orderings is proved by the error in the excitation energy shown in Figure 3 being in the order of eV, that is, of the same order of magnitude as the point-nucleus excitation energies themselves (Table 5). However, one can also observe from Figure 3 that already the value *a* = 4 appears to afford errors in the order of , that is, sub-meV level precision for excitation energies.

### 4.2 Accuracy of Ionization Potentials

**Figure 4:**
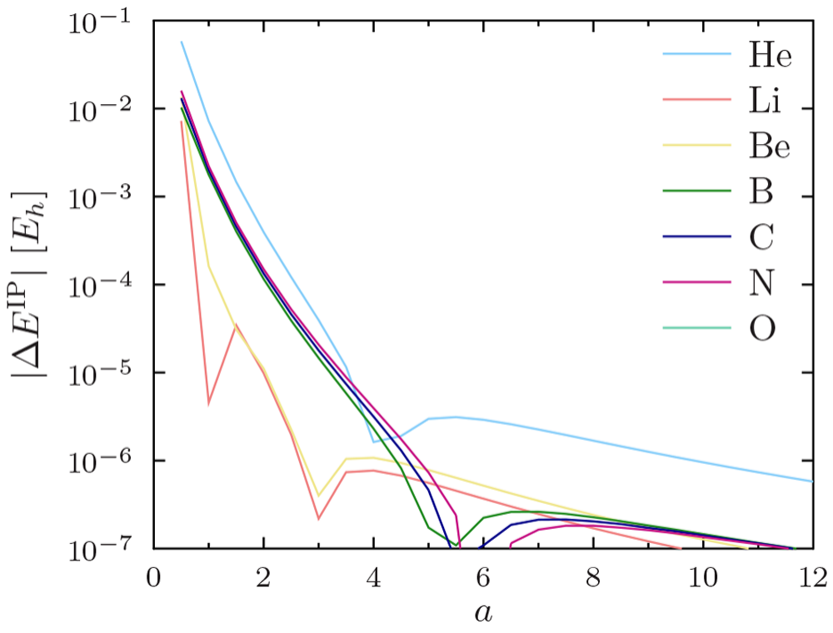
Regularization errors in the PBE ionization potential for He–O as a function of the regularization parameter *a*. Note the use of a logarithmic *y* axis.

**Figure 5:**
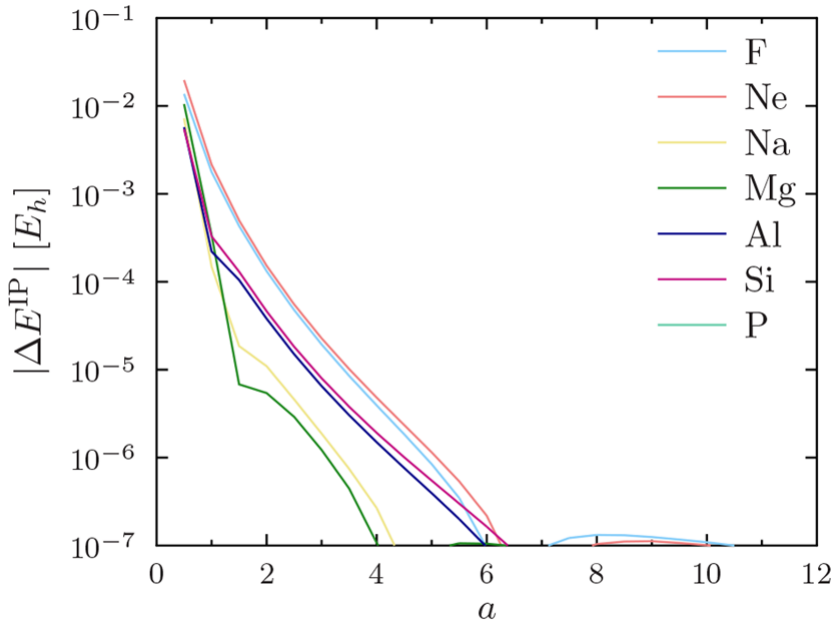
Regularization errors in the PBE ionization potential for F–P as a function of the regularization parameter *a*. Note the use of a logarithmic *y* axis.

**Figure 6:**
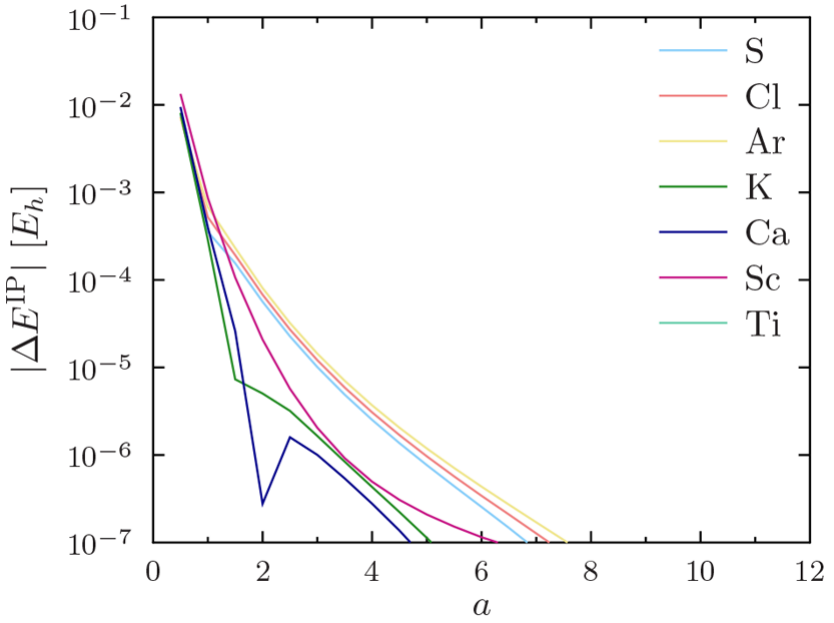
Regularization errors in the PBE ionization potential for S–Ti as a function of the regularization parameter *a*. Note the use of a logarithmic *y* axis.

**Figure 7:**
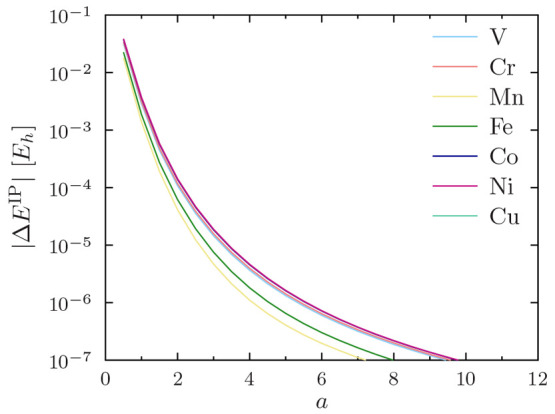
Regularization errors in the PBE ionization potential for V–Cu as a function of the regularization parameter *a*. Note the use of a logarithmic *y* axis.

**Figure 8:**
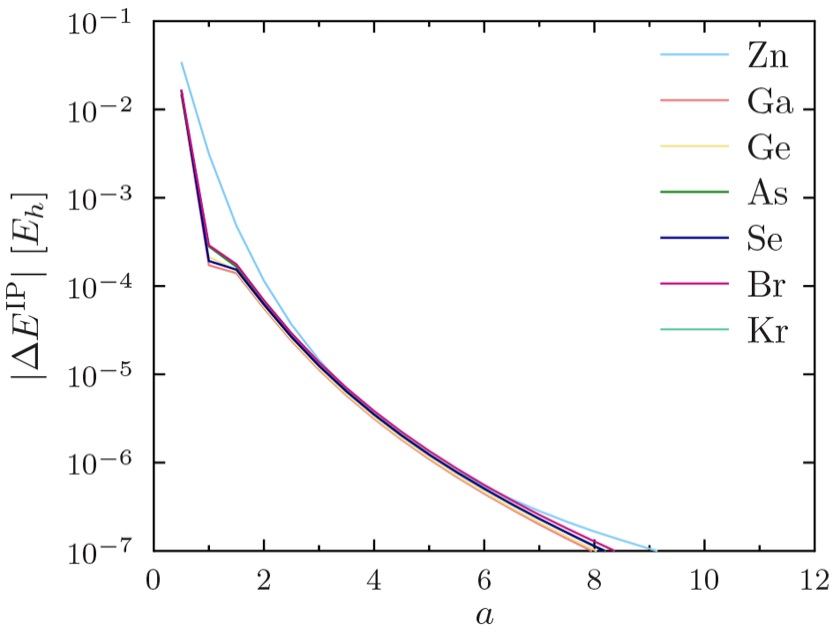
Regularization errors in the PBE ionization potential for Zn–Kr as a function of the regularization parameter *a*. Note the use of a logarithmic *y* axis.

Having established the accuracy of *s*-*d* excitation energies, we continue by examining the accuracy of ionization potentials for He–Kr. Also these calculations employ 25 radial elements. Analogously to section 4.1, we employ a spin-unrestricted formalism with spherically symmetric densities to compute the ionization potential
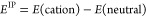
12The errors in the ionization potential
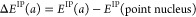
13are shown for the PBE functional in Figure 4 for He–O, in Figure 5 for F–P, in Figure 6 for S–Ti, in Figure 7 for V–Cu, and in Figure 8 for Zn–Kr. The other studied functionals (PW92, TASKCC, and r^2^SCAN) again yielded similar results (not shown).

The ionization potential for the helium atom converges surprisingly slowly with increasing *a*. However, this is easily understood, as the ionization potential of He is really a core property: it depends explicitly on the 1s orbital. The ionization potentials of heavier atoms converge more rapidly to sub-*μE*_*h*_ precision.

One can again observe in Figures 4 to 8 that *a* = 4 affords sub-meV precision of  for ionization potentials in all cases, including He.

## 5 Summary and Conclusions

We have thoroughly examined the regularized nuclear potential recently suggested by Gygi.^19^ We have discussed the determination of the *b* parameter in the potential based on the strength *a* of the regularization and described a simple method to determine values of *b*(*a*) that satisfy the normalization condition to machine precision. We have implemented the potential with this procedure in the HelFEM program,^17,18,22−24^ which we used to carry out a series of atomic calculations to sub-*μE*_*h*_ precision with the PW92, PBE, TASKCC, and r^2^SCAN functionals.

We studied the convergence of total energies, orbital energies, and orbital shapes of closed-shell atoms from Ne to Xe, as well as *s*-*d* excitation energies of Sc–Cu and the ionization potentials of He–Kr. We found that although the total energies converge slowly with *a*, exhibiting differences from the point nucleus value of the order of 0.1 m*E*_*h*_ with *a* = 19, orbital energies and shapes converge much more rapidly, exhibiting small errors already with *a* = 5. The *s*-*d* excitation energies and ionization potentials likewise showed much faster convergence to the point nucleus limit with increasing *a* than the total energies, reaching sub-meV precision with *a* = 4.

These results lend independent support to the accuracy of Gygi’s regularized potential. Although the regularized potential can result in nonmonotonic convergence with respect to *a*, as demonstrated by total energies that can either overestimate or underestimate the point-nucleus value, the rapidity at which many observables converge to the point nucleus values suggests that the regularized potential indeed appears to offer a tractable and reliable way to approach all-electron calculations with plane-waves.

## Supporting Information Available

Convergence plots and tables of orbital energies and positions of orbital electron density maxima for all studied atoms and all studied functionals (PDF)
