# Supplementary material for: Accuracy of a Recent Regularized Nuclear Potential
Source: J Chem Theory Comput. 2023 Jun 24;19(13):4033–9. doi: 10.1021/acs.jctc.3c00530 (PMC10339670; doi:10.1021/acs.jctc.3c00530)
Supplement: Supplementary file 1 — ct3c00530_si_001.pdf [file ct3c00530_si_001.pdf]

Supporting Information: On the accuracy of a recent regularized nuclear potential

Susi Lehtola*,†

Department of Chemistry, University of Helsinki, P.O. Box 55, FI-00014 University of Helsinki, Finland

E-mail: susi.lehtola@alumni.helsinki.fi

First, we show the full set of regularization errors in atomic total energies. Positive energy differences ($E^{\text{regularized}} > E^{\text{Coulomb}}$) are shown with blue squares and negative energy differences ($E^{\text{regularized}} < E^{\text{Coulomb}}$) with red triangles in all figures.

- He atom: HF in fig. S1, PW92 in fig. S2, PBE in fig. S3, TASKCC in fig. S4.
- Be atom: HF in fig. S5, PW92 in fig. S6, PBE in fig. S7, TASKCC in fig. S8.
- Ne atom: HF in fig. S9, PW92 in fig. S10, PBE in fig. S11, TASKCC in fig. S12.
- Mg atom: HF in fig. S13, PW92 in fig. S14, PBE in fig. S15, TASKCC in fig. S16.
- Ar atom: HF in fig. S17, PW92 in fig. S18, PBE in fig. S19, TASKCC in fig. S20.
- Ca atom: HF in fig. S21, PW92 in fig. S22, PBE in fig. S23, TASKCC in fig. S24.
- Zn atom: HF in fig. S25, PW92 in fig. S26, PBE in fig. S27, TASKCC in fig. S28.
- Kr atom: HF in fig. S29, PW92 in fig. S30, PBE in fig. S31, TASKCC in fig. S32.
- Sr atom: HF in fig. S33, PW92 in fig. S34, PBE in fig. S35, TASKCC in fig. S36.
- Cd atom: HF in fig. S37, PW92 in fig. S38, PBE in fig. S39, TASKCC in fig. S40.
- Xe atom: HF in fig. S41, PW92 in fig. S42, PBE in fig. S43, TASKCC in fig. S44.

Then, we also show the regularization errors in orbital energies and positions of orbital density maxima for all calculations:

- He atom: HF in tables S1 and S2, respectively, PW92 in tables S3 and S4, respectively, PBE in tables S5 and S6, respectively, TASKCC in tables S7 and S8, respectively.
- Be atom: HF in tables S9 and S10, respectively, PW92 in tables S11 and S12, respectively, PBE in tables S13 and S14, respectively, TASKCC in tables S15 and S16, respectively.
- Ne atom: HF in tables S17 and S18, respectively, PW92 in tables S19 and S20, respectively, PBE in tables S21 and S22, respectively, TASKCC in tables S23 and S24, respectively.
- Mg atom: HF in tables S25 and S26, respectively, PW92 in tables S27 and S28, respectively, PBE in tables S29 and S30, respectively, TASKCC in tables S31 and S32, respectively.

- Ar atom: HF in tables S33 and S34, respectively, PW92 in tables S35 and S36, respectively, PBE in tables S37 and S38, respectively, TASKCC in tables S39 and S40, respectively.

- Ca atom: HF in tables S41 and S42, respectively, PW92 in tables S43 and S44, respectively, PBE in tables S45 and S46, respectively, TASKCC in tables S47 and S48, respectively.

- Zn atom: HF in tables S49 and S50, respectively, PW92 in tables S51 and S52, respectively, PBE in tables S53 and S54, respectively, TASKCC in tables S55 and S56, respectively.

- Kr atom: HF in tables S57 and S58, respectively, PW92 in tables S59 and S60, respectively, PBE in tables S61 and S62, respectively, TASKCC in tables S63 and S64, respectively.

- Sr atom: HF in tables S65 and S66, respectively, PW92 in tables S67 and S68, respectively, PBE in tables S69 and S70, respectively, TASKCC in tables S71 and S72, respectively.

- Cd atom: HF in tables S73 and S74, respectively, PW92 in tables S75 and S76, respectively, PBE in tables S77 and S78, respectively, TASKCC in tables S79 and S80, respectively.

- Xe atom: HF in tables S81 and S82, respectively, PW92 in tables S83 and S84, respectively, PBE in tables S85 and S86, respectively, TASKCC in tables S87 and S88, respectively.

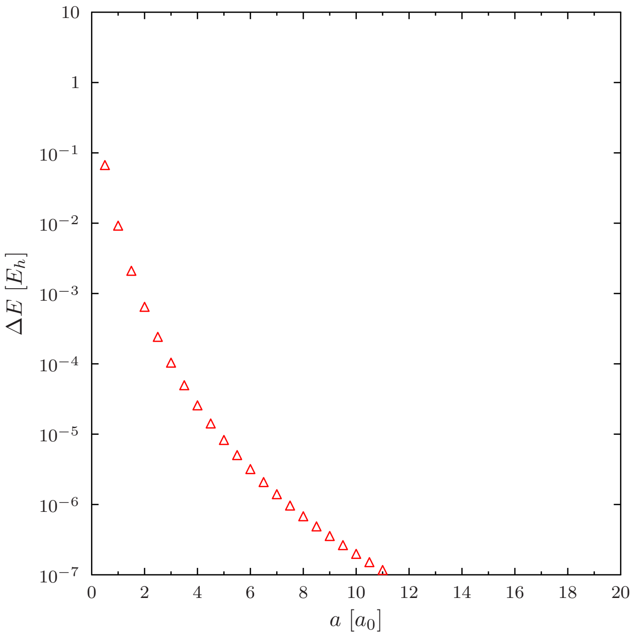

Figure S1: Regularization error in the total energy of the He atom calculated with HF.

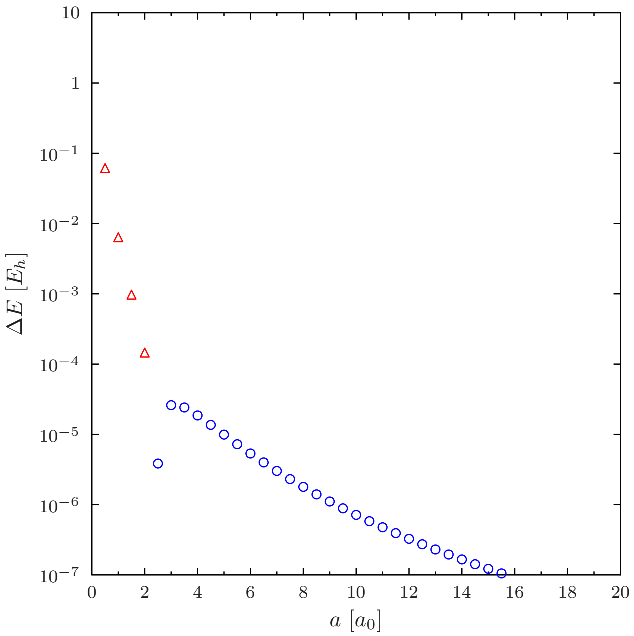

Figure S2: Regularization error in the total energy of the He atom calculated with PW92.

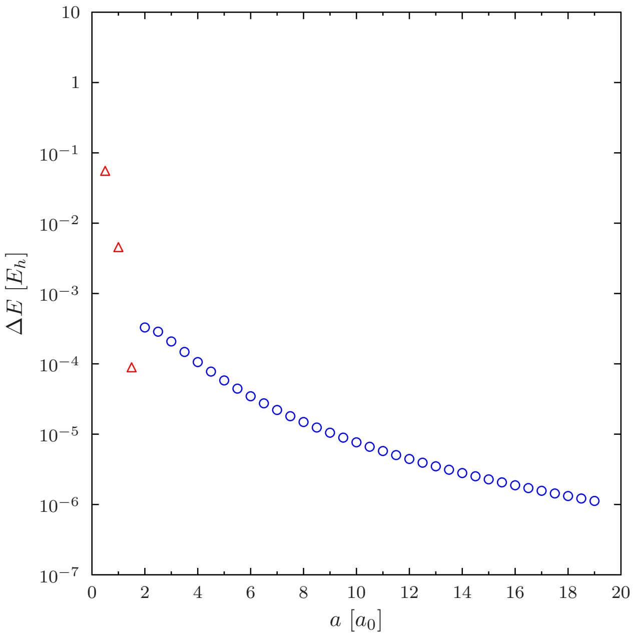

Figure S3: Regularization error in the total energy of the He atom calculated with PBE.

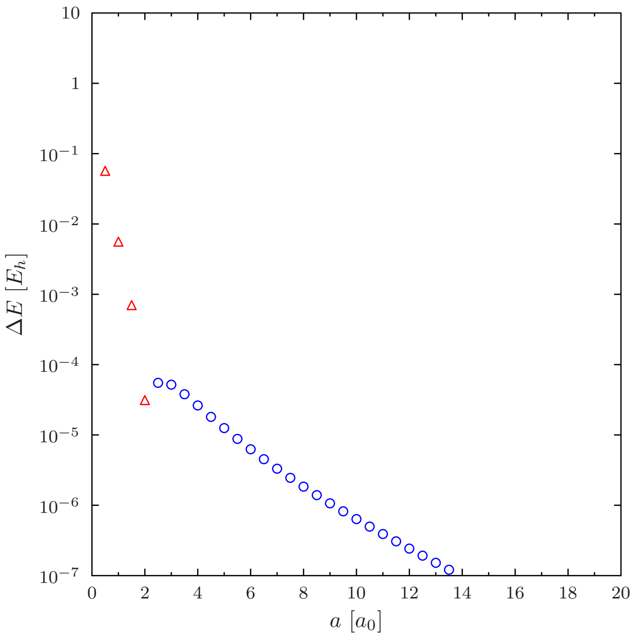

Figure S4: Regularization error in the total energy of the He atom calculated with TASKCC.

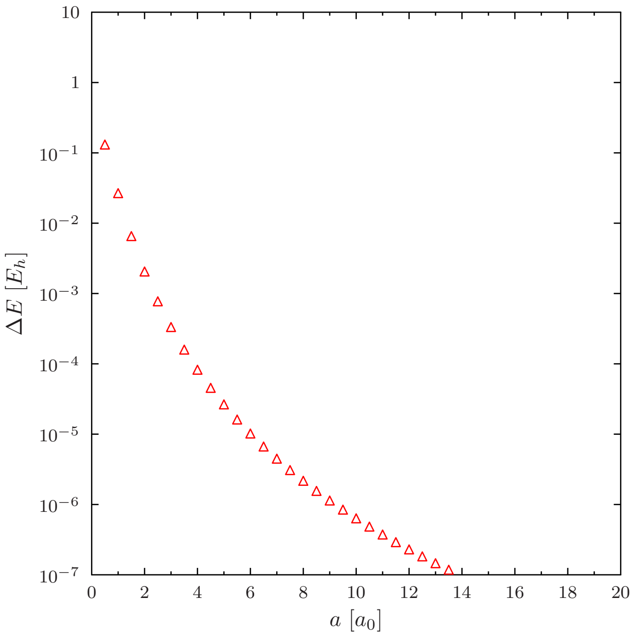

Figure S5: Regularization error in the total energy of the Be atom calculated with HF.

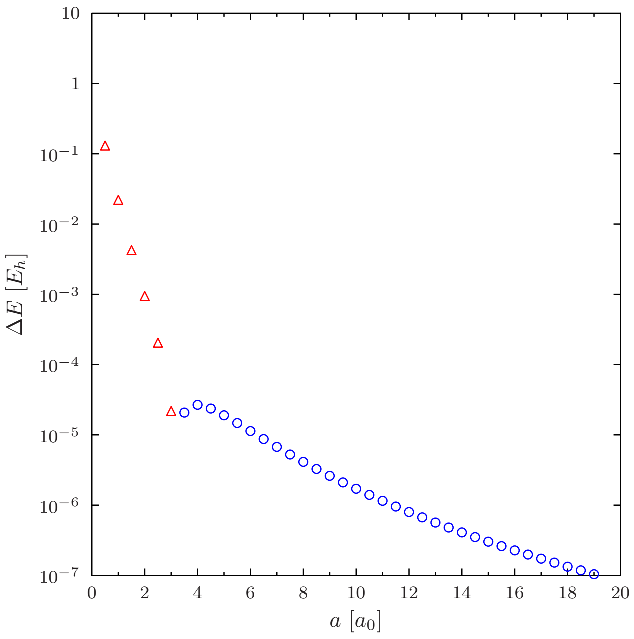

Figure S6: Regularization error in the total energy of the Be atom calculated with PW92.

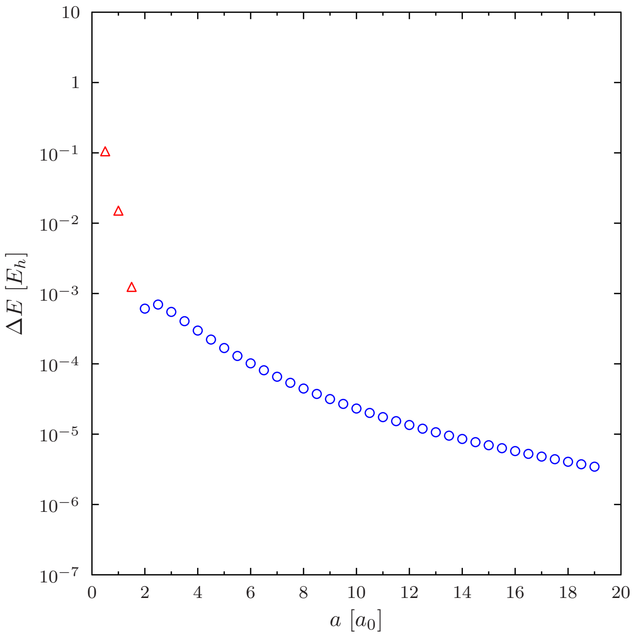

Figure S7: Regularization error in the total energy of the Be atom calculated with PBE.

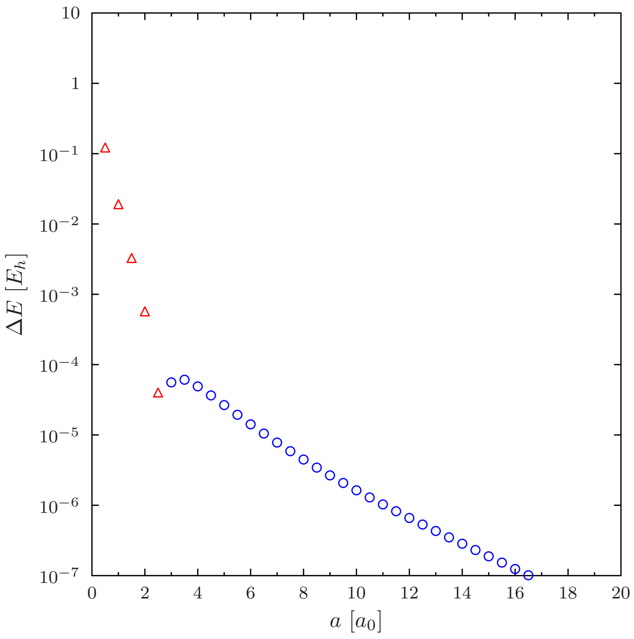

Figure S8: Regularization error in the total energy of the Be atom calculated with TASKCC.

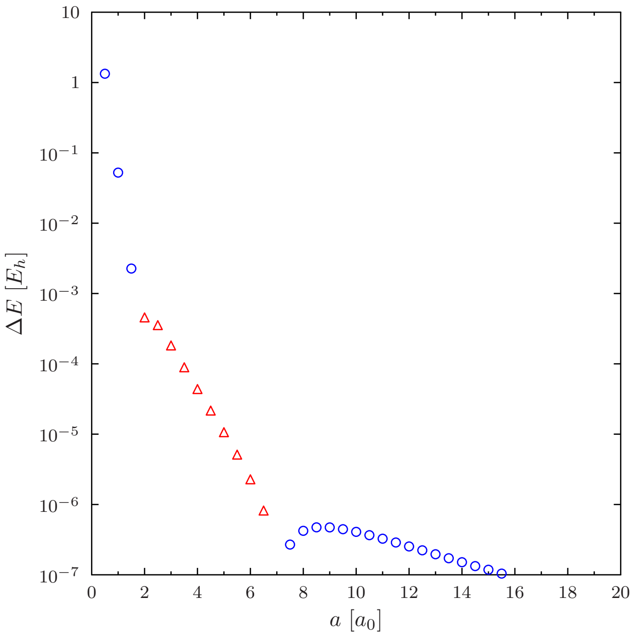

Figure S9: Regularization error in the total energy of the Ne atom calculated with HF.

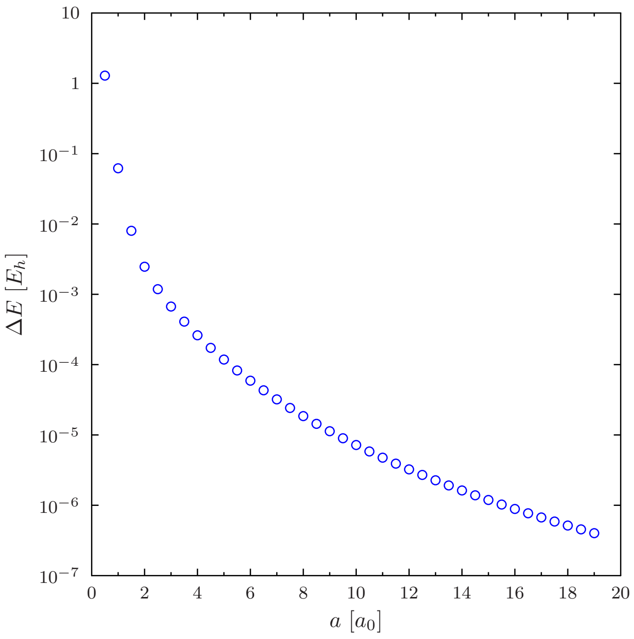

Figure S10: Regularization error in the total energy of the Ne atom calculated with PW92.

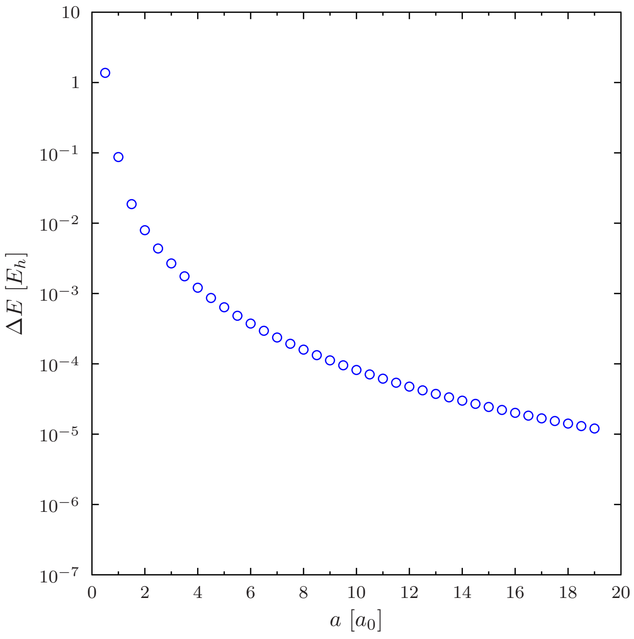

Figure S11: Regularization error in the total energy of the Ne atom calculated with PBE.

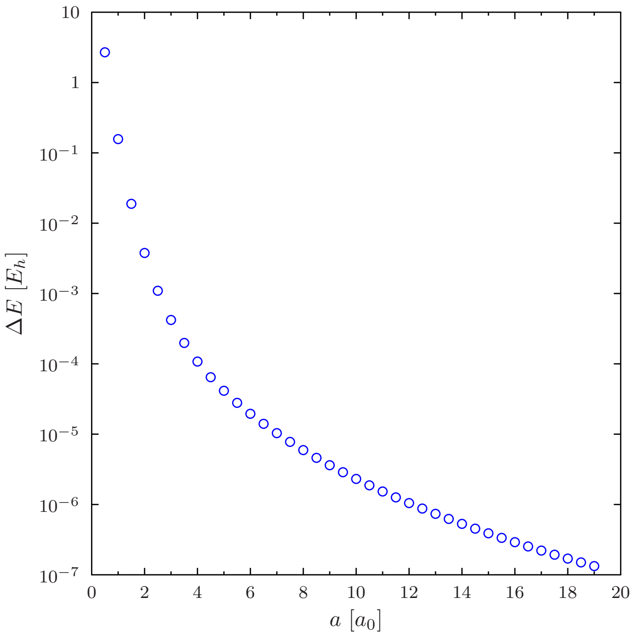

Figure S13: Regularization error in the total energy of the Mg atom calculated with HF.

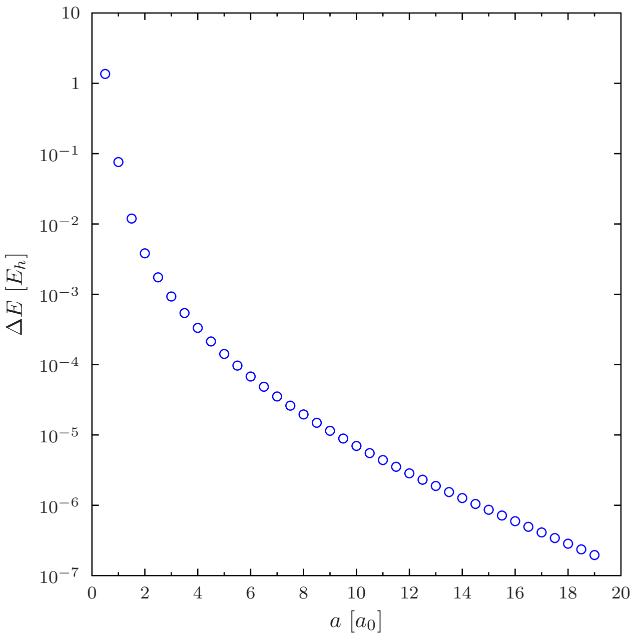

Figure S12: Regularization error in the total energy of the Ne atom calculated with TASKCC.

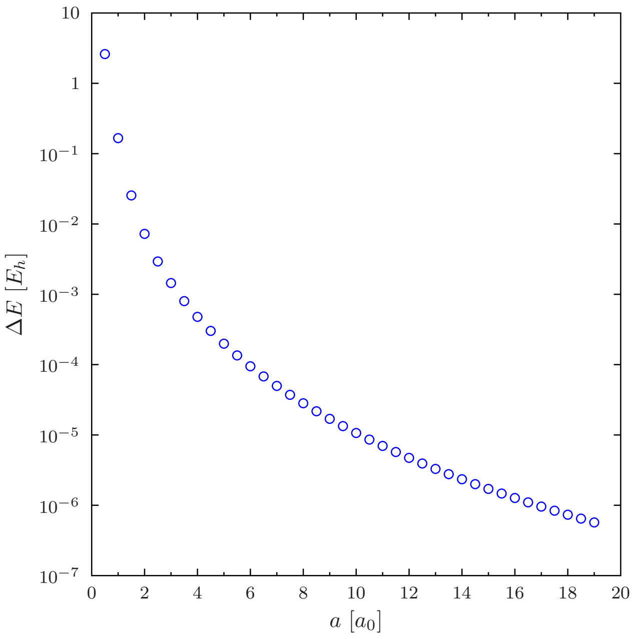

Figure S14: Regularization error in the total energy of the Mg atom calculated with PW92.

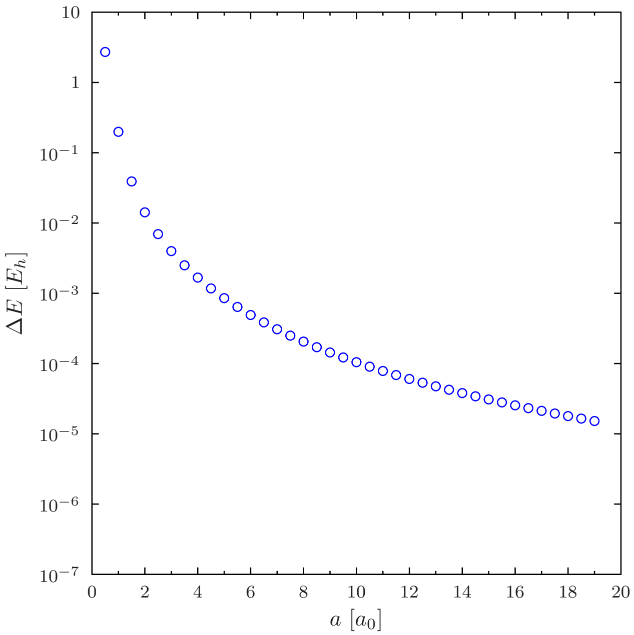

Figure S15: Regularization error in the total energy of the Mg atom calculated with PBE.

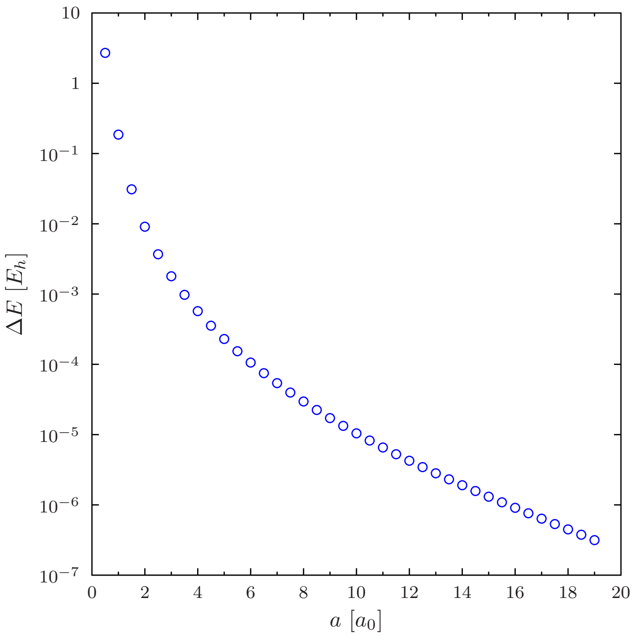

Figure S16: Regularization error in the total energy of the Mg atom calculated with TASKCC.

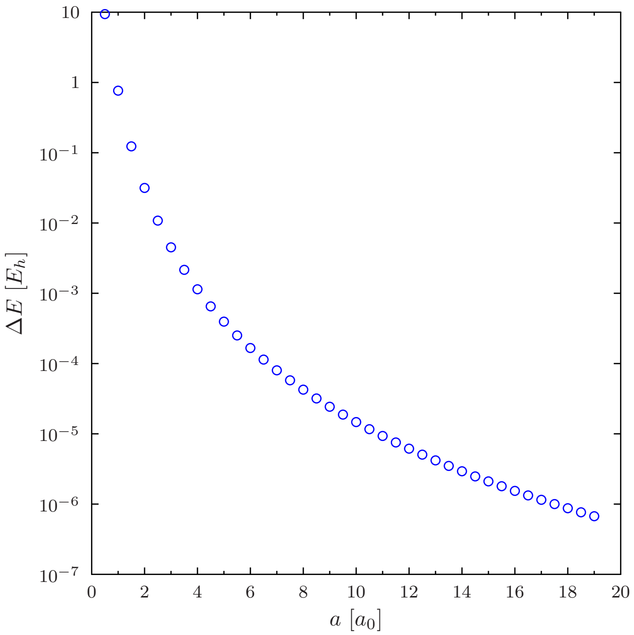

Figure S17: Regularization error in the total energy of the Ar atom calculated with HF.

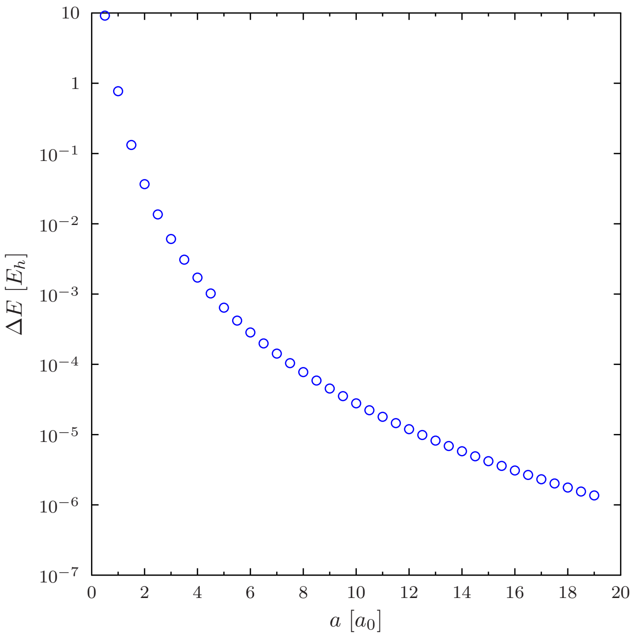

Figure S18: Regularization error in the total energy of the Ar atom calculated with PW92.

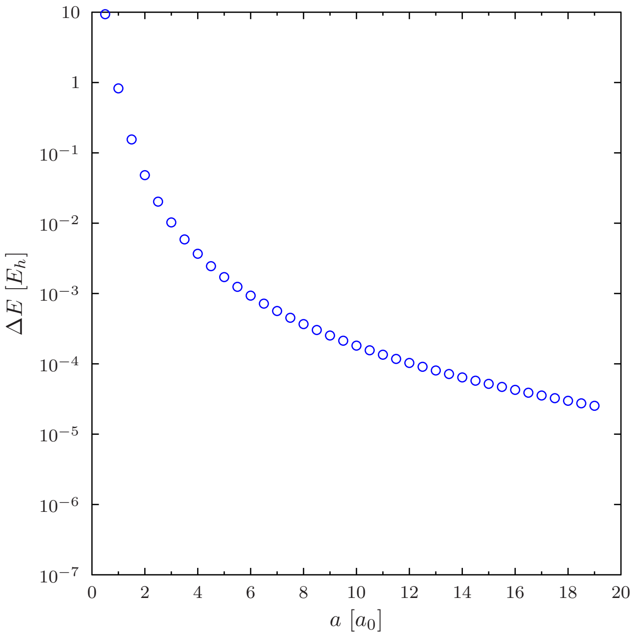

Figure S19: Regularization error in the total energy of the Ar atom calculated with PBE.

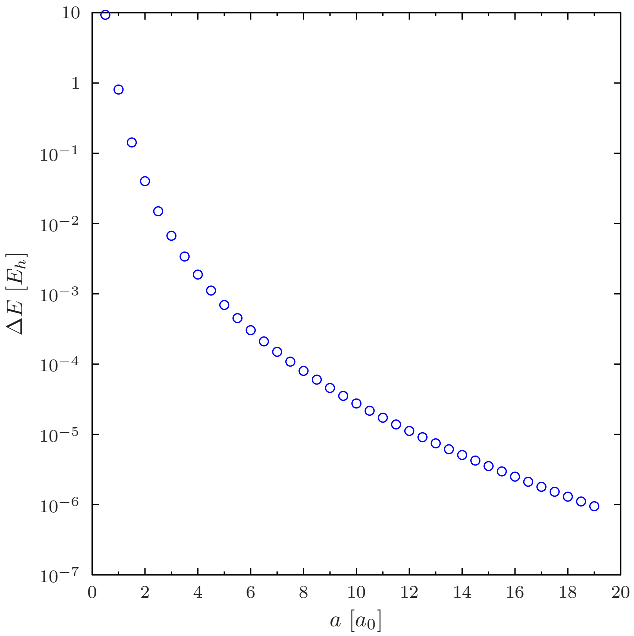

Figure S20: Regularization error in the total energy of the Ar atom calculated with TASKCC.

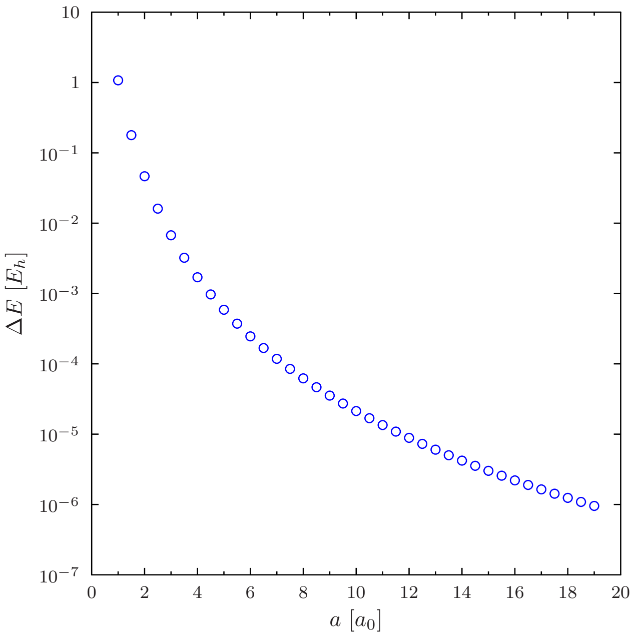

Figure S21: Regularization error in the total energy of the Ca atom calculated with HF.

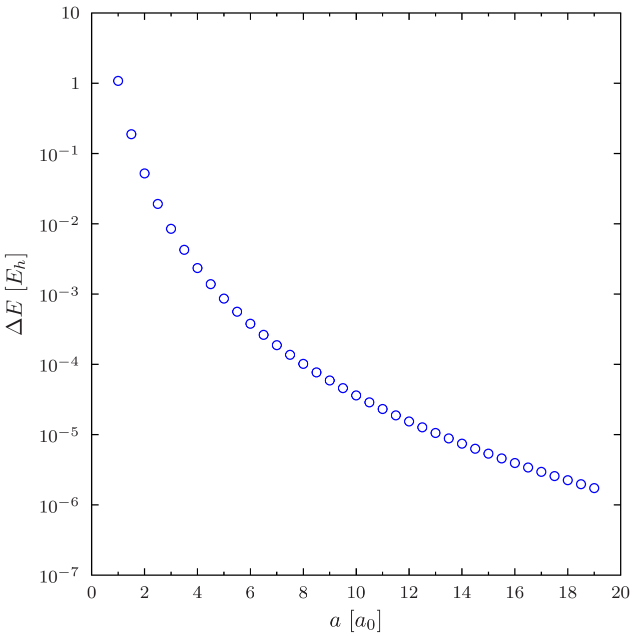

Figure S22: Regularization error in the total energy of the Ca atom calculated with PW92.

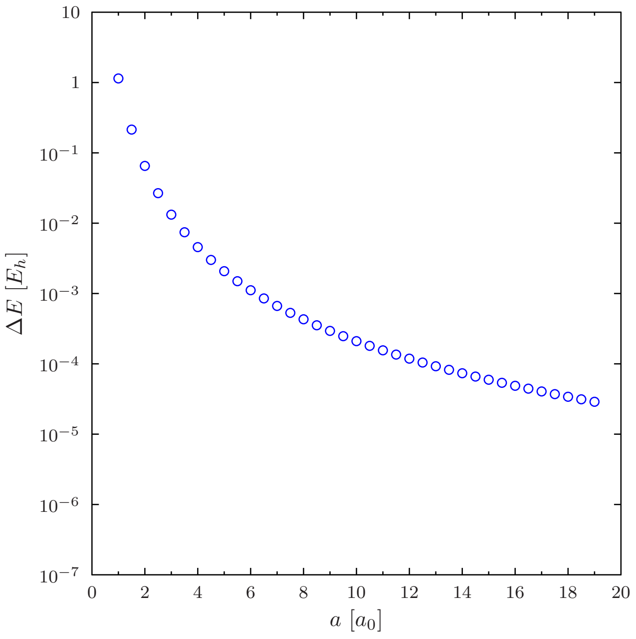

Figure S23: Regularization error in the total energy of the Ca atom calculated with PBE.

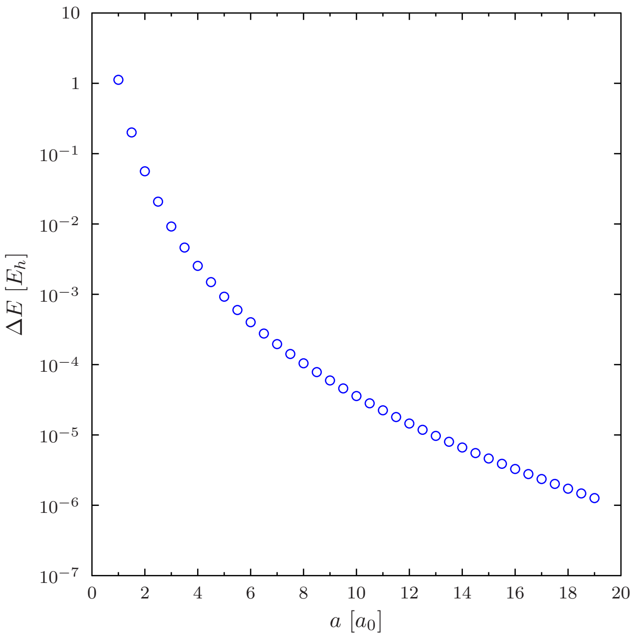

Figure S24: Regularization error in the total energy of the Ca atom calculated with TASKCC.

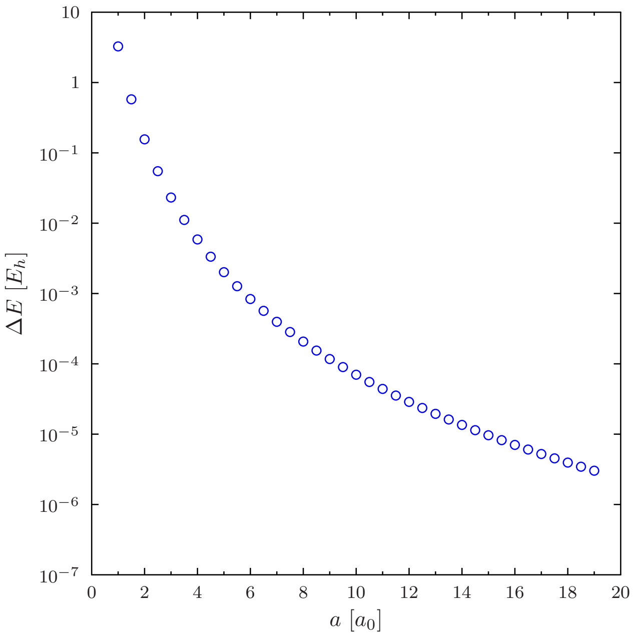

Figure S25: Regularization error in the total energy of the Zn atom calculated with HF.

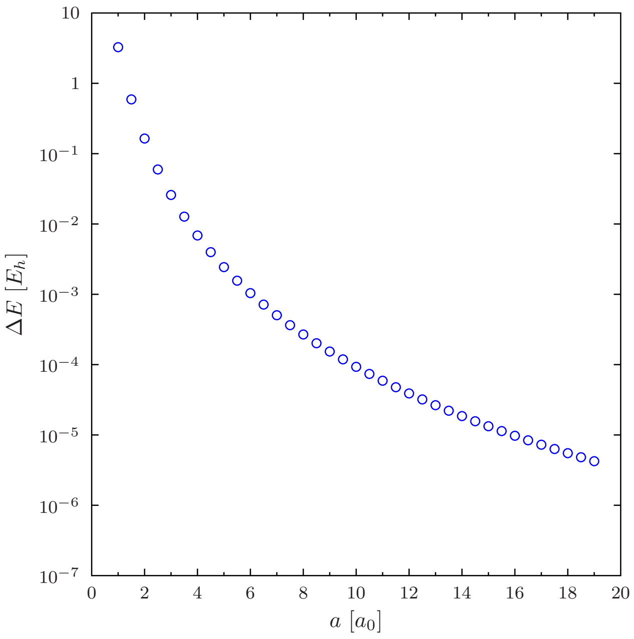

Figure S26: Regularization error in the total energy of the Zn atom calculated with PW92.

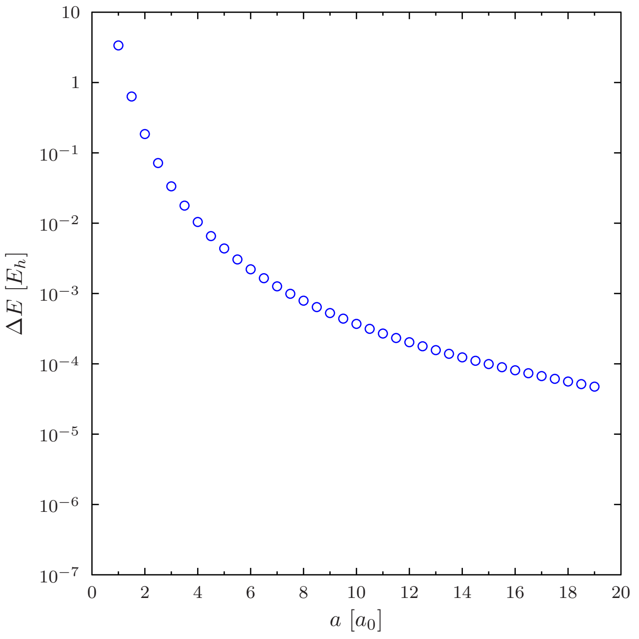

Figure S27: Regularization error in the total energy of the Zn atom calculated with PBE.

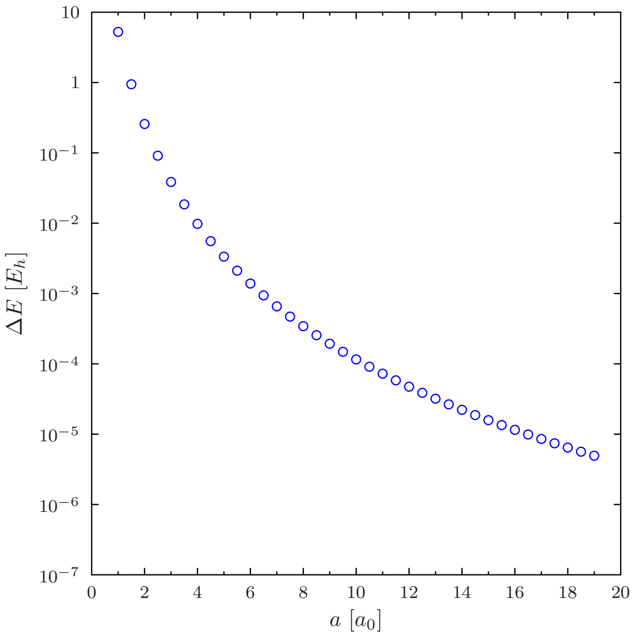

Figure S29: Regularization error in the total energy of the Kr atom calculated with HF.

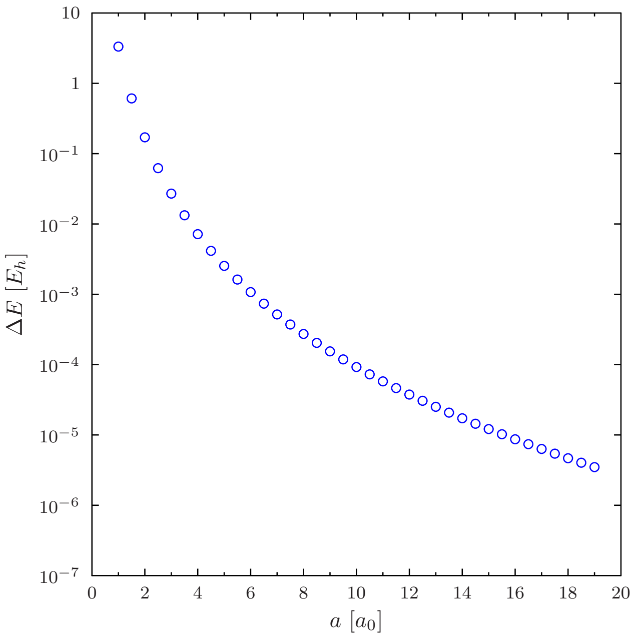

Figure S28: Regularization error in the total energy of the Zn atom calculated with TASKCC.

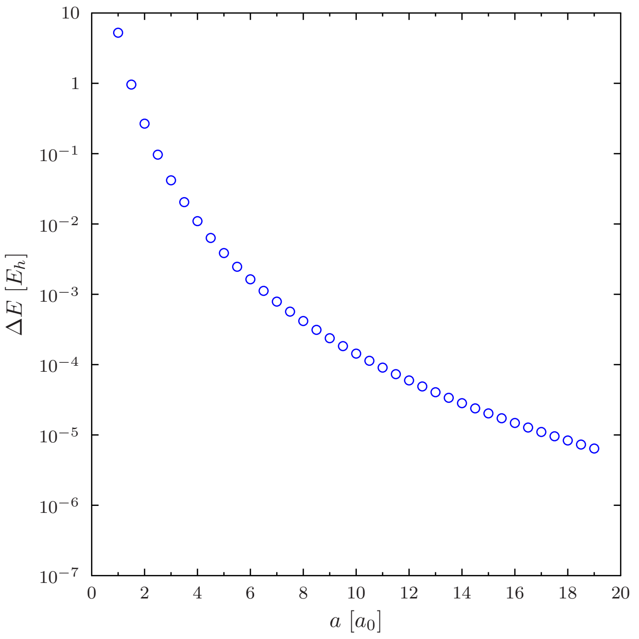

Figure S30: Regularization error in the total energy of the Kr atom calculated with PW92.

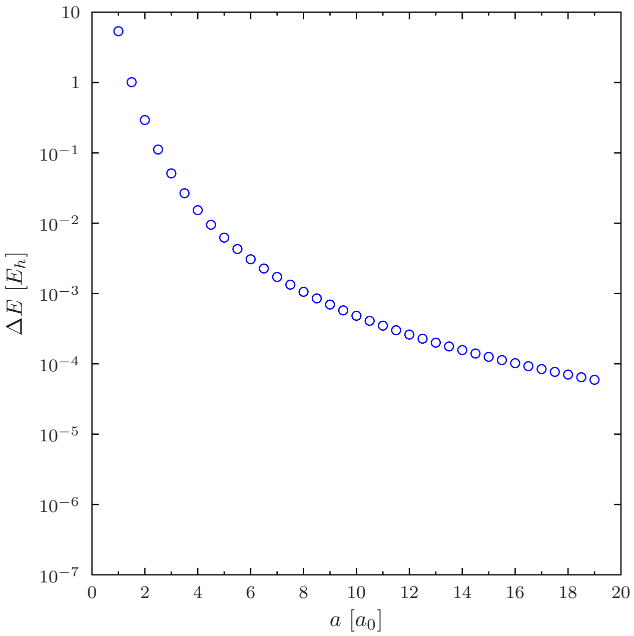

Figure S31: Regularization error in the total energy of the Kr atom calculated with PBE.

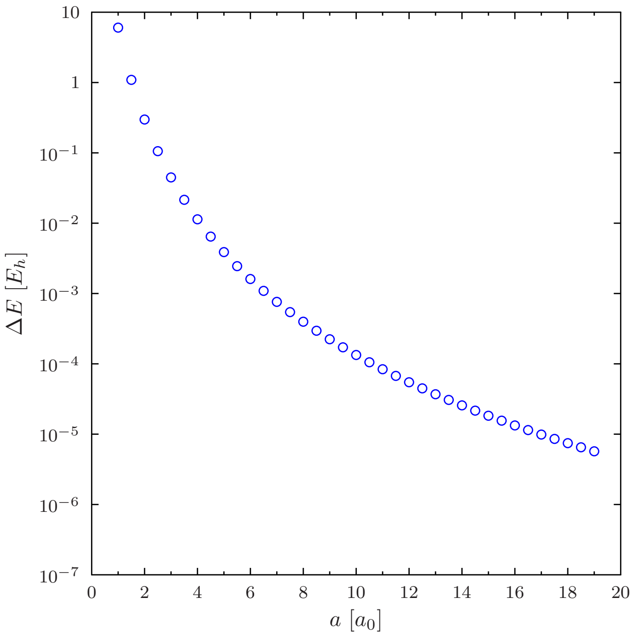

Figure S33: Regularization error in the total energy of the Sr atom calculated with HF.

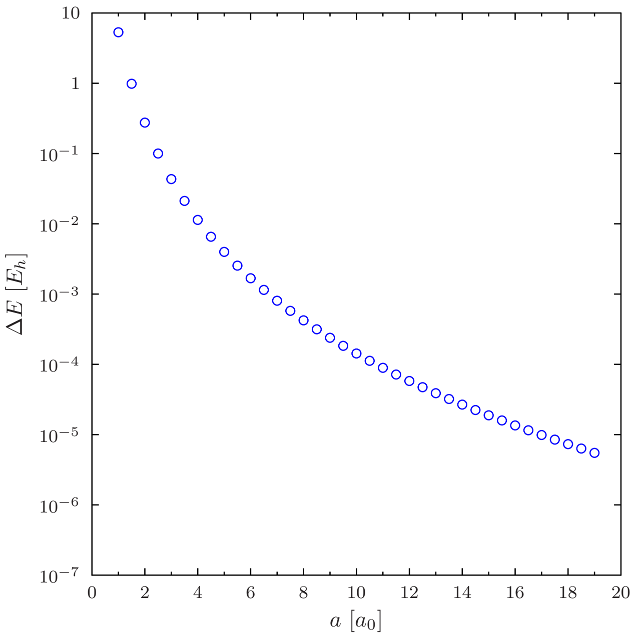

Figure S32: Regularization error in the total energy of the Kr atom calculated with TASKCC.

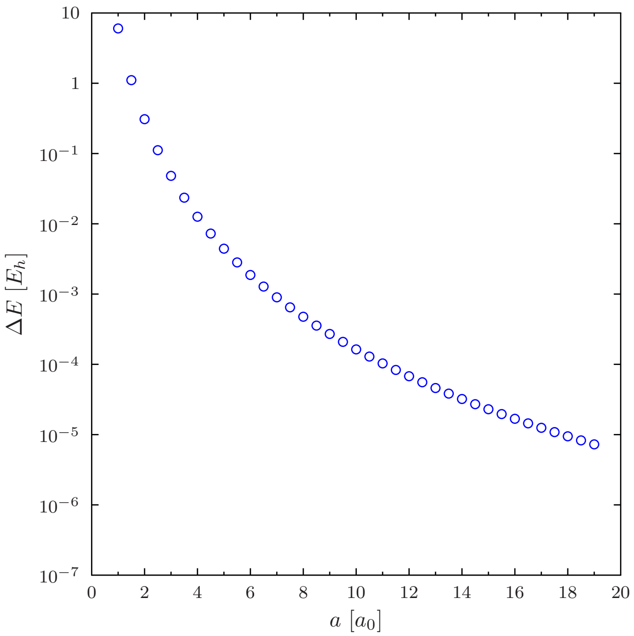

Figure S34: Regularization error in the total energy of the Sr atom calculated with PW92.

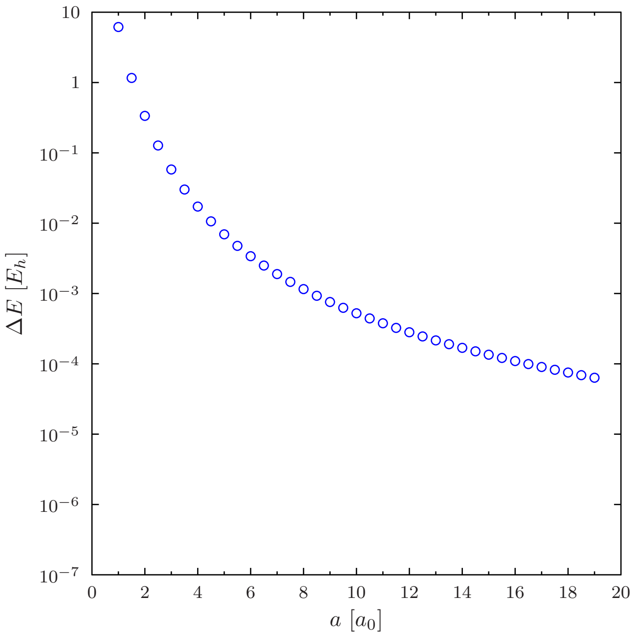

Figure S35: Regularization error in the total energy of the Sr atom calculated with PBE.

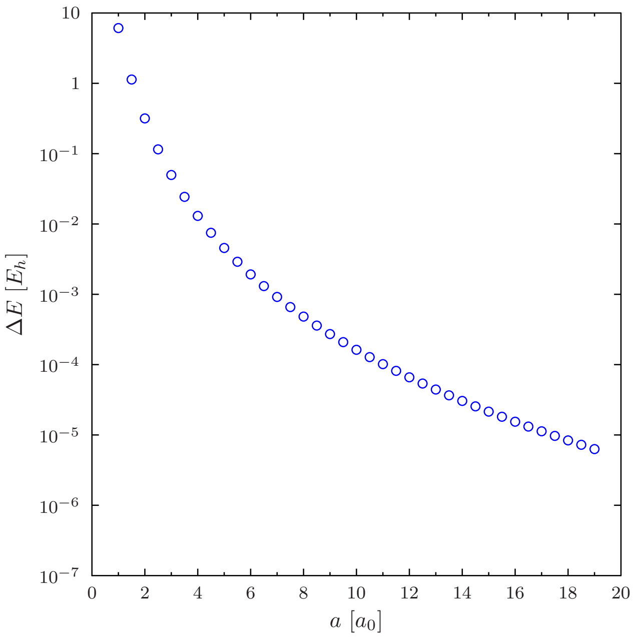

Figure S36: Regularization error in the total energy of the Sr atom calculated with TASKCC.

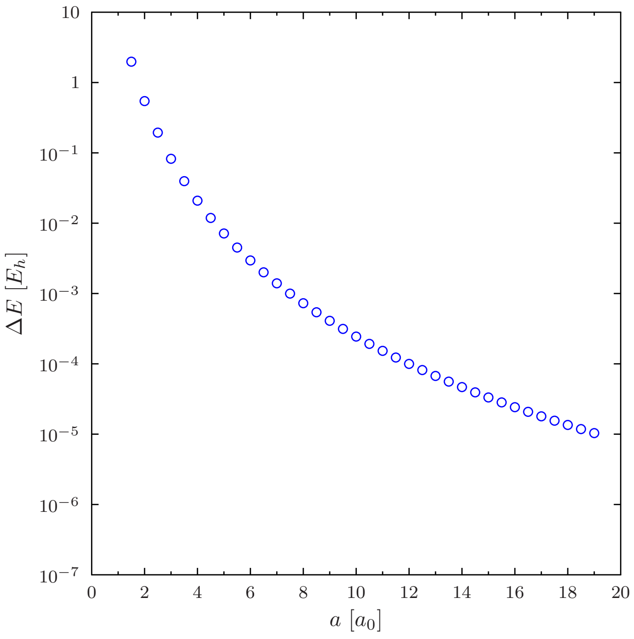

Figure S37: Regularization error in the total energy of the Cd atom calculated with HF.

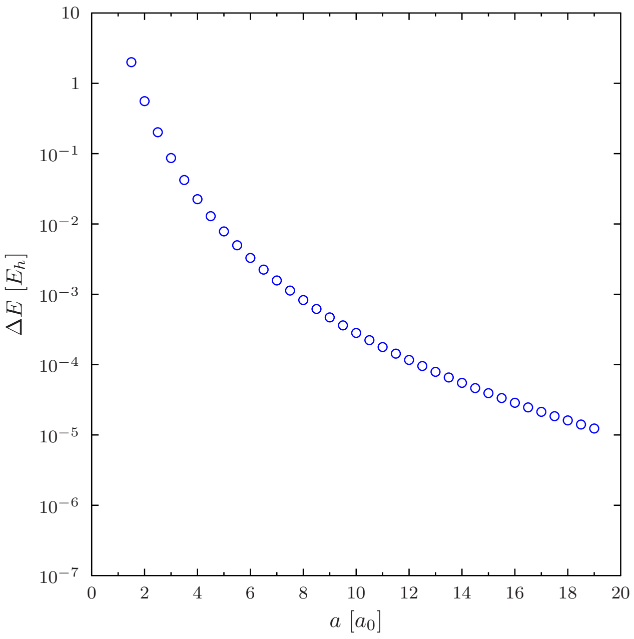

Figure S38: Regularization error in the total energy of the Cd atom calculated with PW92.

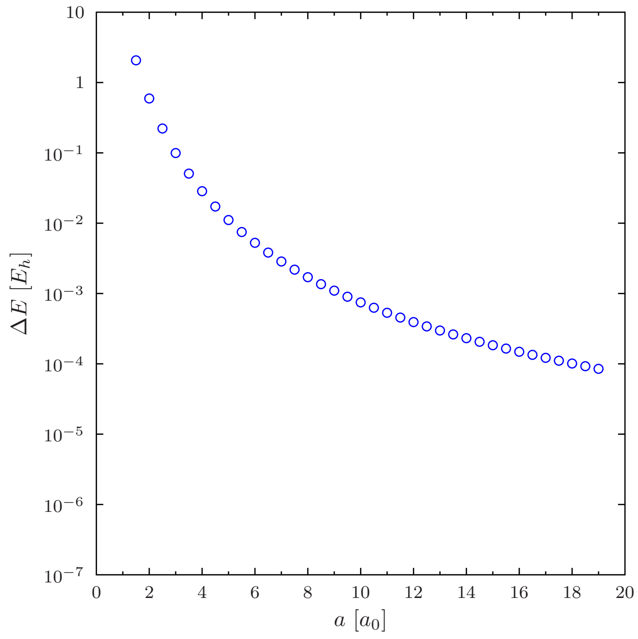

Figure S39: Regularization error in the total energy of the Cd atom calculated with PBE.

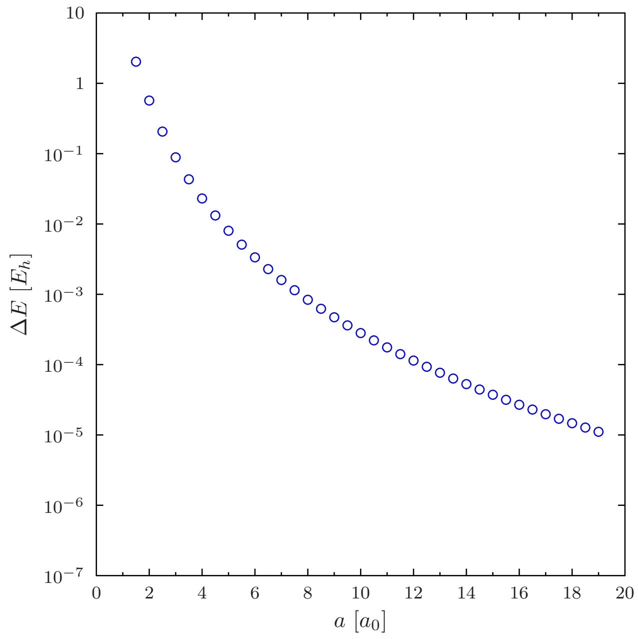

Figure S40: Regularization error in the total energy of the Cd atom calculated with TASKCC.

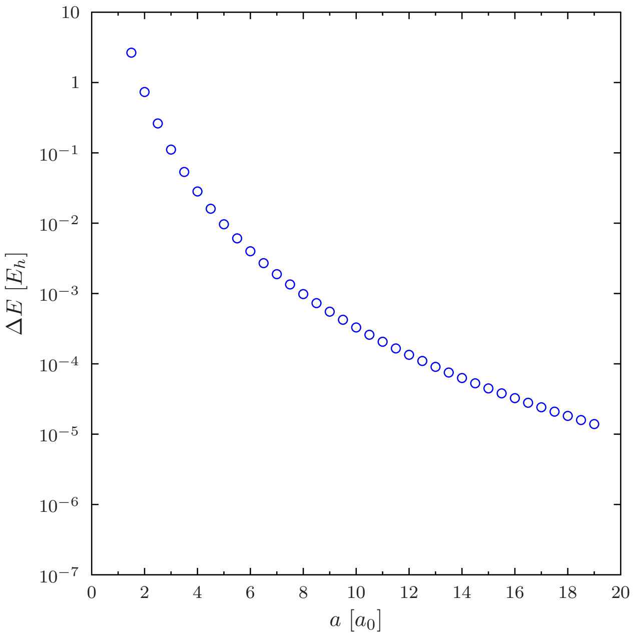

Figure S41: Regularization error in the total energy of the Xe atom calculated with HF.

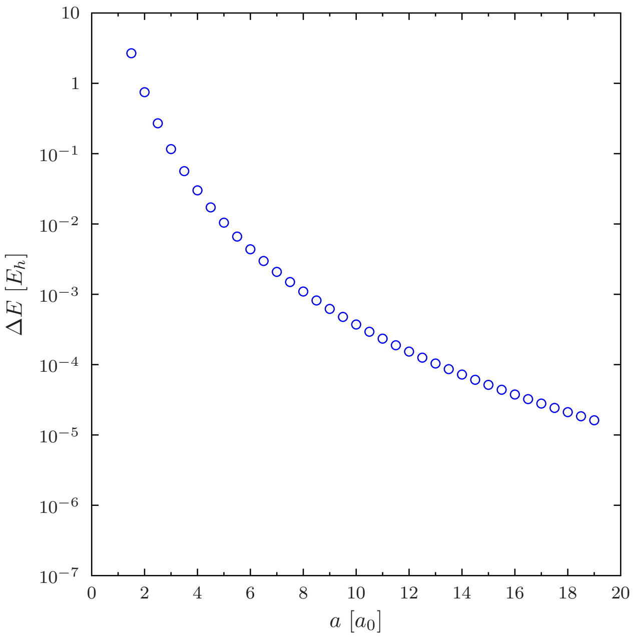

Figure S42: Regularization error in the total energy of the Xe atom calculated with PW92.

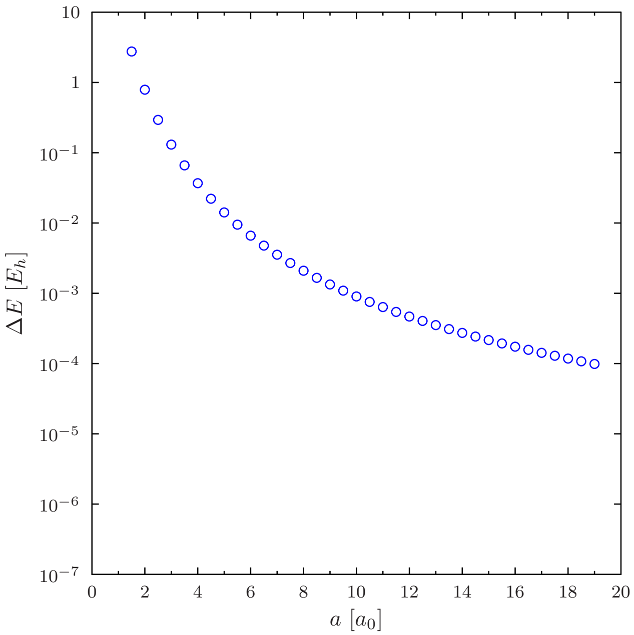

Figure S43: Regularization error in the total energy of the Xe atom calculated with PBE.

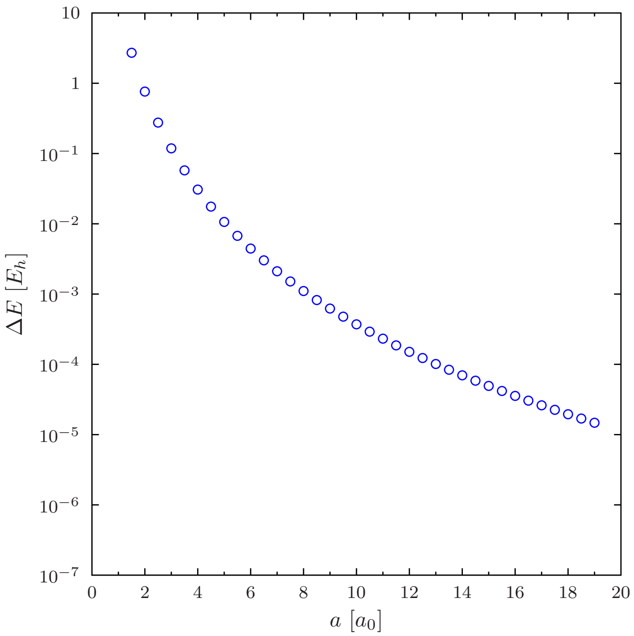

Figure S44: Regularization error in the total energy of the Xe atom calculated with TASKCC.

Table S1: Errors in orbital energies in E_h for the He atom computed with HF and the regularized potential with various values of a. The values obtained with the Coulomb potential of the point nucleus are shown in the last column. For comparison, the last row shows the errors in total energy ΔE from the point nucleus value shown in the last column.

Energy	$a = 1.0$	$a = 2.0$	$a = 3.0$	$a = 5.0$	$a = 7.0$	point nucleus
$1s$	−0.0052	−0.0003	−0.0001	−0.0000	−0.0000	−0.9180
ΔE	−0.0088312	−0.0006190	−0.0000995	−0.0000079	−0.0000013	−2.8616800

Table S2: Errors in positions of orbital density maxima in bohr for the He atom computed with HF and the regularized potential with various values of a. The values obtained with the Coulomb potential of the point nucleus are shown in the last column.

Energy	$a = 1.0$	$a = 2.0$	$a = 3.0$	$a = 5.0$	$a = 7.0$	point nucleus
$1s$	−0.016784	−0.002700	−0.000052	−0.000004	−0.000000	0.569145

Table S3: Errors in orbital energies in E_h for the He atom computed with PW92 and the regularized potential with various values of a. The values obtained with the Coulomb potential of the point nucleus are shown in the last column. For comparison, the last row shows the errors in total energy ΔE from the point nucleus value shown in the last column.

Energy	$a = 1.0$	$a = 2.0$	$a = 3.0$	$a = 5.0$	$a = 7.0$	point nucleus
$1s$	−0.0036	−0.0002	−0.0000	0.0000	0.0000	−0.5703
ΔE	−0.0061111	−0.0001397	0.0000260	0.0000099	0.0000030	−2.8344552

Table S4: Errors in positions of orbital density maxima in bohr for the He atom computed with PW92 and the regularized potential with various values of a. The values obtained with the Coulomb potential of the point nucleus are shown in the last column.

Energy	$a = 1.0$	$a = 2.0$	$a = 3.0$	$a = 5.0$	$a = 7.0$	point nucleus
$1s$	−0.016881	−0.002732	−0.000032	0.000001	0.000001	0.569641

Table S5: Errors in orbital energies in E_h for the He atom computed with PBE and the regularized potential with various values of a. The values obtained with the Coulomb potential of the point nucleus are shown in the last column. For comparison, the last row shows the errors in total energy ΔE from the point nucleus value shown in the last column.

Energy	$a = 1.0$	$a = 2.0$	$a = 3.0$	$a = 5.0$	$a = 7.0$	point nucleus
$1s$	−0.0040	−0.0001	−0.0000	0.0000	0.0000	−0.5793
ΔE	−0.0043708	0.0003289	0.0002079	0.0000581	0.0000221	−2.8929349

Table S6: Errors in positions of orbital density maxima in bohr for the He atom computed with PBE and the regularized potential with various values of a. The values obtained with the Coulomb potential of the point nucleus are shown in the last column.

Energy	$a = 1.0$	$a = 2.0$	$a = 3.0$	$a = 5.0$	$a = 7.0$	point nucleus
$1s$	−0.014547	−0.003028	−0.000016	0.000006	0.000003	0.560260

Table S7: Errors in orbital energies in E_h for the He atom computed with TASKCC and the regularized potential with various values of a. The values obtained with the Coulomb potential of the point nucleus are shown in the last column. For comparison, the last row shows the errors in total energy ΔE from the point nucleus value shown in the last column.

Energy	$a = 1.0$	$a = 2.0$	$a = 3.0$	$a = 5.0$	$a = 7.0$	point nucleus
$1s$	−0.0042	−0.0002	−0.0000	0.0000	0.0000	−0.6426
ΔE	−0.0053662	−0.0000300	0.0000518	0.0000125	0.0000033	−2.9794485

Table S8: Errors in positions of orbital density maxima in bohr for the He atom computed with TASKCC and the regularized potential with various values of a. The values obtained with the Coulomb potential of the point nucleus are shown in the last column.

Energy	$a = 1.0$	$a = 2.0$	$a = 3.0$	$a = 5.0$	$a = 7.0$	point nucleus
$1s$	−0.013521	−0.002950	−0.000027	0.000002	0.000001	0.559246

Table S9: Errors in orbital energies in E_h for the Be atom computed with HF and the regularized potential with various values of a. The values obtained with the Coulomb potential of the point nucleus are shown in the last column. For comparison, the last row shows the errors in total energy ΔE from the point nucleus value shown in the last column.

Energy	$a = 1.0$	$a = 2.0$	$a = 3.0$	$a = 5.0$	$a = 7.0$	point nucleus
$1s$	−0.0236	−0.0015	−0.0002	−0.0000	−0.0000	−4.7327
$2s$	0.0001	−0.0000	−0.0000	−0.0000	−0.0000	−0.3093
ΔE	−0.0255920	−0.0019684	−0.0003195	−0.0000254	−0.0000043	−14.5730232

Table S10: Errors in positions of orbital density maxima in bohr for the Be atom computed with HF and the regularized potential with various values of a. The values obtained with the Coulomb potential of the point nucleus are shown in the last column.

Energy	$a = 1.0$	$a = 2.0$	$a = 3.0$	$a = 5.0$	$a = 7.0$	point nucleus
$1s$	−0.003048	−0.001723	−0.000024	−0.000001	−0.000000	0.267051
$2s$	0.000854	−0.000197	−0.000038	−0.000003	0.000000	2.050591

Table S11: Errors in orbital energies in E_h for the Be atom computed with PW92 and the regularized potential with various values of a. The values obtained with the Coulomb potential of the point nucleus are shown in the last column. For comparison, the last row shows the errors in total energy ΔE from the point nucleus value shown in the last column.

Energy	$a = 1.0$	$a = 2.0$	$a = 3.0$	$a = 5.0$	$a = 7.0$	point nucleus
$1s$	−0.0255	−0.0013	−0.0001	−0.0000	0.0000	−3.8561
$2s$	−0.0000	−0.0000	−0.0000	−0.0000	0.0000	−0.2058
ΔE	−0.0211475	−0.0009060	−0.0000210	0.0000190	0.0000067	−14.4464735

Table S12: Errors in positions of orbital density maxima in bohr for the Be atom computed with PW92 and the regularized potential with various values of a. The values obtained with the Coulomb potential of the point nucleus are shown in the last column.

Energy	$a = 1.0$	$a = 2.0$	$a = 3.0$	$a = 5.0$	$a = 7.0$	point nucleus
$1s$	−0.003532	−0.001701	−0.000020	−0.000000	0.000000	0.268766
$2s$	0.000747	−0.000150	−0.000020	0.000000	0.000000	2.041544

Table S13: Errors in orbital energies in E_h for the Be atom computed with PBE and the regularized potential with various values of a. The values obtained with the Coulomb potential of the point nucleus are shown in the last column. For comparison, the last row shows the errors in total energy ΔE from the point nucleus value shown in the last column.

Energy	$a = 1.0$	$a = 2.0$	$a = 3.0$	$a = 5.0$	$a = 7.0$	point nucleus
1s	−0.0259	−0.0011	−0.0001	0.0000	0.0000	−3.9026
2s	0.0000	−0.0000	−0.0000	0.0000	0.0000	−0.2061
ΔE	−0.0144345	0.0006093	0.0005475	0.0001677	0.0000655	−14.6299477

Table S14: Errors in positions of orbital density maxima in bohr for the Be atom computed with PBE and the regularized potential with various values of a. The values obtained with the Coulomb potential of the point nucleus are shown in the last column.

Energy	$a = 1.0$	$a = 2.0$	$a = 3.0$	$a = 5.0$	$a = 7.0$	point nucleus
1s	−0.002851	−0.001780	−0.000015	0.000002	0.000001	0.266176
2s	0.001050	−0.000090	0.000003	0.000006	0.000002	2.042185

Table S15: Errors in orbital energies in E_h for the Be atom computed with TASKCC and the regularized potential with various values of a. The values obtained with the Coulomb potential of the point nucleus are shown in the last column. For comparison, the last row shows the errors in total energy ΔE from the point nucleus value shown in the last column.

Energy	$a = 1.0$	$a = 2.0$	$a = 3.0$	$a = 5.0$	$a = 7.0$	point nucleus
1s	−0.0249	−0.0012	−0.0001	0.0000	0.0000	−4.0563
2s	−0.0000	−0.0000	−0.0000	0.0000	0.0000	−0.2253
ΔE	−0.0183023	−0.0005482	0.0000559	0.0000266	0.0000078	−14.7471259

Table S16: Errors in positions of orbital density maxima in bohr for the Be atom computed with TASKCC and the regularized potential with various values of a. The values obtained with the Coulomb potential of the point nucleus are shown in the last column.

Energy	$a = 1.0$	$a = 2.0$	$a = 3.0$	$a = 5.0$	$a = 7.0$	point nucleus
1s	−0.002540	−0.001765	−0.000019	0.000000	0.000000	0.265586
2s	0.000682	−0.000123	−0.000012	0.000001	0.000001	2.108092

Table S17: Errors in orbital energies in E_h for the Ne atom computed with HF and the regularized potential with various values of a. The values obtained with the Coulomb potential of the point nucleus are shown in the last column. For comparison, the last row shows the errors in total energy ΔE from the point nucleus value shown in the last column.

Energy	$a = 1.0$	$a = 2.0$	$a = 3.0$	$a = 5.0$	$a = 7.0$	point nucleus
1s	−0.1358	−0.0087	−0.0014	−0.0001	−0.0000	−32.7724
2s	0.0002	−0.0006	−0.0001	−0.0000	−0.0000	−1.9304
2p	0.0026	0.0002	0.0000	0.0000	0.0000	−0.8504
ΔE	0.0523800	−0.0004377	−0.0001747	−0.0000102	−0.0000001	−128.5470981

Table S18: Errors in positions of orbital density maxima in bohr for the Ne atom computed with HF and the regularized potential with various values of a. The values obtained with the Coulomb potential of the point nucleus are shown in the last column.

Energy	$a = 1.0$	$a = 2.0$	$a = 3.0$	$a = 5.0$	$a = 7.0$	point nucleus
1s	−0.000052	−0.000767	−0.000009	−0.000000	−0.000000	0.102961
2s	0.001802	−0.000016	−0.000008	−0.000001	−0.000000	0.682866
2p	0.004276	0.000218	0.000034	0.000003	0.000001	0.634034

Table S19: Errors in orbital energies in E_h for the Ne atom computed with PW92 and the regularized potential with various values of a. The values obtained with the Coulomb potential of the point nucleus are shown in the last column. For comparison, the last row shows the errors in total energy ΔE from the point nucleus value shown in the last column.

Energy	$a = 1.0$	$a = 2.0$	$a = 3.0$	$a = 5.0$	$a = 7.0$	point nucleus
1s	−0.1538	−0.0089	−0.0012	−0.0001	−0.0000	−30.3058
2s	−0.0016	−0.0005	−0.0001	−0.0000	−0.0000	−1.3226
2p	0.0012	0.0001	0.0000	0.0000	0.0000	−0.4978
ΔE	0.0619536	0.0024657	0.0006697	0.0001180	0.0000320	−128.2299172

Table S20: Errors in positions of orbital density maxima in bohr for the Ne atom computed with PW92 and the regularized potential with various values of a. The values obtained with the Coulomb potential of the point nucleus are shown in the last column.

Energy	$a = 1.0$	$a = 2.0$	$a = 3.0$	$a = 5.0$	$a = 7.0$	point nucleus
1s	−0.000147	−0.000763	−0.000009	−0.000000	0.000000	0.103303
2s	0.001621	−0.000022	−0.000007	−0.000001	−0.000000	0.684641
2p	0.004359	0.000225	0.000035	0.000003	0.000001	0.619414

Table S21: Errors in orbital energies in E_h for the Ne atom computed with PBE and the regularized potential with various values of a. The values obtained with the Coulomb potential of the point nucleus are shown in the last column. For comparison, the last row shows the errors in total energy ΔE from the point nucleus value shown in the last column.

Energy	$a = 1.0$	$a = 2.0$	$a = 3.0$	$a = 5.0$	$a = 7.0$	point nucleus
1s	−0.1494	−0.0077	−0.0007	0.0001	0.0000	−30.4893
2s	−0.0008	−0.0004	−0.0001	−0.0000	0.0000	−1.3332
2p	0.0011	0.0001	0.0000	0.0000	−0.0000	−0.4905
ΔE	0.0871932	0.0079460	0.0026781	0.0006372	0.0002368	−128.8664277

Table S22: Errors in positions of orbital density maxima in bohr for the Ne atom computed with PBE and the regularized potential with various values of a. The values obtained with the Coulomb potential of the point nucleus are shown in the last column.

Energy	$a = 1.0$	$a = 2.0$	$a = 3.0$	$a = 5.0$	$a = 7.0$	point nucleus
$1s$	−0.000025	−0.000775	−0.000008	0.000000	0.000000	0.102852
$2s$	0.001786	0.000001	0.000000	0.000001	0.000001	0.682308
$2p$	0.004281	0.000218	0.000033	0.000003	0.000000	0.622366

Table S23: Errors in orbital energies in E_h for the Ne atom computed with TASKCC and the regularized potential with various values of a. The values obtained with the Coulomb potential of the point nucleus are shown in the last column. For comparison, the last row shows the errors in total energy ΔE from the point nucleus value shown in the last column.

Energy	$a = 1.0$	$a = 2.0$	$a = 3.0$	$a = 5.0$	$a = 7.0$	point nucleus
$1s$	−0.1474	−0.0083	−0.0011	−0.0000	−0.0000	−30.7918
$2s$	−0.0013	−0.0005	−0.0001	−0.0000	−0.0000	−1.4406
$2p$	0.0013	0.0001	0.0000	0.0000	0.0000	−0.5317
ΔE	0.0759949	0.0038248	0.0009306	0.0001417	0.0000352	−128.9847733

Table S24: Errors in positions of orbital density maxima in bohr for the Ne atom computed with TASKCC and the regularized potential with various values of a. The values obtained with the Coulomb potential of the point nucleus are shown in the last column.

Energy	$a = 1.0$	$a = 2.0$	$a = 3.0$	$a = 5.0$	$a = 7.0$	point nucleus
$1s$	−0.000008	−0.000772	−0.000009	0.000000	0.000000	0.102846
$2s$	0.001726	−0.000009	−0.000004	−0.000000	0.000000	0.682910
$2p$	0.004459	0.000231	0.000035	0.000003	0.000001	0.627850

Table S25: Errors in orbital energies in E_h for the Mg atom computed with HF and the regularized potential with various values of a. The values obtained with the Coulomb potential of the point nucleus are shown in the last column. For comparison, the last row shows the errors in total energy ΔE from the point nucleus value shown in the last column.

Energy	$a = 1.0$	$a = 2.0$	$a = 3.0$	$a = 5.0$	$a = 7.0$	point nucleus
$1s$	−0.1803	−0.0115	−0.0018	−0.0001	−0.0000	−49.0317
$2s$	0.0067	−0.0005	−0.0001	−0.0000	−0.0000	−3.7677
$2p$	0.0092	0.0006	0.0001	0.0000	0.0000	−2.2822
$3s$	0.0004	−0.0000	−0.0000	−0.0000	−0.0000	−0.2531
ΔE	0.1568757	0.0037706	0.0004204	0.0000414	0.0000103	−199.6146364

Table S26: Errors in positions of orbital density maxima in bohr for the Mg atom computed with HF and the regularized potential with various values of a. The values obtained with the Coulomb potential of the point nucleus are shown in the last column.

Energy	$a = 1.0$	$a = 2.0$	$a = 3.0$	$a = 5.0$	$a = 7.0$	point nucleus
$1s$	0.000060	−0.000645	−0.000008	−0.000000	−0.000000	0.085455
$2s$	0.001786	0.000008	−0.000003	−0.000000	−0.000000	0.546392
$2p$	0.003382	0.000174	0.000027	0.000002	0.000000	0.483857
$3s$	0.004245	−0.000077	−0.000029	−0.000004	−0.000001	2.585818

Table S27: Errors in orbital energies in E_h for the Mg atom computed with PW92 and the regularized potential with various values of a. The values obtained with the Coulomb potential of the point nucleus are shown in the last column. For comparison, the last row shows the errors in total energy ΔE from the point nucleus value shown in the last column.

Energy	$a = 1.0$	$a = 2.0$	$a = 3.0$	$a = 5.0$	$a = 7.0$	point nucleus
$1s$	−0.2048	−0.0119	−0.0016	−0.0001	−0.0000	−45.9730
$2s$	0.0038	−0.0005	−0.0001	−0.0000	−0.0000	−2.9035
$2p$	0.0069	0.0005	0.0001	0.0000	0.0000	−1.7187
$3s$	0.0002	−0.0000	−0.0000	−0.0000	−0.0000	−0.1755
ΔE	0.1661910	0.0072293	0.0014451	0.0001984	0.0000498	−199.1352883

Table S28: Errors in positions of orbital density maxima in bohr for the Mg atom computed with PW92 and the regularized potential with various values of a. The values obtained with the Coulomb potential of the point nucleus are shown in the last column.

Energy	$a = 1.0$	$a = 2.0$	$a = 3.0$	$a = 5.0$	$a = 7.0$	point nucleus
$1s$	−0.000008	−0.000643	−0.000007	−0.000000	0.000000	0.085699
$2s$	0.001663	0.000003	−0.000003	−0.000000	0.000000	0.549917
$2p$	0.003411	0.000177	0.000027	0.000002	0.000001	0.477654
$3s$	0.003501	−0.000110	−0.000030	−0.000003	0.000000	2.518348

Table S29: Errors in orbital energies in E_h for the Mg atom computed with PBE and the regularized potential with various values of a. The values obtained with the Coulomb potential of the point nucleus are shown in the last column. For comparison, the last row shows the errors in total energy ΔE from the point nucleus value shown in the last column.

Energy	$a = 1.0$	$a = 2.0$	$a = 3.0$	$a = 5.0$	$a = 7.0$	point nucleus
$1s$	−0.1988	−0.0104	−0.0010	0.0001	0.0001	−46.2009
$2s$	0.0046	−0.0004	−0.0001	0.0000	0.0000	−2.9235
$2p$	0.0065	0.0005	0.0001	0.0000	0.0000	−1.7146
$3s$	0.0002	−0.0000	−0.0000	0.0000	0.0000	−0.1727
ΔE	0.1985448	0.0141840	0.0039820	0.0008526	0.0003076	−199.9551152

Table S30: Errors in positions of orbital density maxima in bohr for the Mg atom computed with PBE and the regularized potential with various values of a. The values obtained with the Coulomb potential of the point nucleus are shown in the last column.

Energy	$a = 1.0$	$a = 2.0$	$a = 3.0$	$a = 5.0$	$a = 7.0$	point nucleus
$1s$	0.000079	−0.000651	−0.000006	0.000000	0.000000	0.085382
$2s$	0.001767	0.000018	0.000002	0.000001	0.000000	0.548158
$2p$	0.003376	0.000174	0.000026	0.000002	0.000000	0.479202
$3s$	0.004042	−0.000051	−0.000012	0.000002	0.000001	2.559083

Table S31: Errors in orbital energies in E_h for the Mg atom computed with TASKCC and the regularized potential with various values of a. The values obtained with the Coulomb potential of the point nucleus are shown in the last column. For comparison, the last row shows the errors in total energy ΔE from the point nucleus value shown in the last column.

Energy	$a = 1.0$	$a = 2.0$	$a = 3.0$	$a = 5.0$	$a = 7.0$	point nucleus
$1s$	−0.1945	−0.0112	−0.0015	−0.0001	−0.0000	−46.6129
$2s$	0.0044	−0.0005	−0.0001	−0.0000	−0.0000	−3.1041
$2p$	0.0072	0.0005	0.0001	0.0000	0.0000	−1.8254
$3s$	0.0002	−0.0000	−0.0000	−0.0000	−0.0000	−0.1849
ΔE	0.1857753	0.0090901	0.0017925	0.0002294	0.0000540	−200.0383120

Table S32: Errors in positions of orbital density maxima in bohr for the Mg atom computed with TASKCC and the regularized potential with various values of a. The values obtained with the Coulomb potential of the point nucleus are shown in the last column.

Energy	$a = 1.0$	$a = 2.0$	$a = 3.0$	$a = 5.0$	$a = 7.0$	point nucleus
$1s$	0.000085	−0.000649	−0.000007	−0.000000	0.000000	0.085395
$2s$	0.001738	0.000012	−0.000001	−0.000000	0.000000	0.548447
$2p$	0.003458	0.000181	0.000028	0.000002	0.000000	0.480783
$3s$	0.004261	−0.000094	−0.000027	−0.000002	0.000000	2.713031

Table S33: Errors in orbital energies in E_h for the Ar atom computed with HF and the regularized potential with various values of a. The values obtained with the Coulomb potential of the point nucleus are shown in the last column. For comparison, the last row shows the errors in total energy ΔE from the point nucleus value shown in the last column.

Energy	$a = 1.0$	$a = 2.0$	$a = 3.0$	$a = 5.0$	$a = 7.0$	point nucleus
$1s$	−0.3248	−0.0214	−0.0034	−0.0003	−0.0001	−118.6104
$2s$	0.0490	−0.0003	−0.0002	−0.0000	−0.0000	−12.3222
$2p$	0.0474	0.0027	0.0004	0.0000	0.0000	−9.5715
$3s$	0.0044	−0.0001	−0.0000	−0.0000	−0.0000	−1.2774
$3p$	0.0008	0.0001	0.0000	0.0000	0.0000	−0.5910
ΔE	0.7641531	0.0315516	0.0045067	0.0003948	0.0000801	−526.8175128

Table S34: Errors in positions of orbital density maxima in bohr for the Ar atom computed with HF and the regularized potential with various values of a. The values obtained with the Coulomb potential of the point nucleus are shown in the last column.

Energy	$a = 1.0$	$a = 2.0$	$a = 3.0$	$a = 5.0$	$a = 7.0$	point nucleus
$1s$	0.000161	−0.000438	−0.000005	−0.000000	−0.000000	0.056567
$2s$	0.001502	0.000028	0.000001	−0.000000	0.000000	0.339084
$2p$	0.002093	0.000111	0.000018	0.000002	0.000000	0.282094
$3s$	0.003746	0.000045	−0.000002	0.000000	0.000000	1.182481
$3p$	0.003496	0.000226	0.000038	0.000004	0.000001	1.299655

Table S35: Errors in orbital energies in E_h for the Ar atom computed with PW92 and the regularized potential with various values of a. The values obtained with the Coulomb potential of the point nucleus are shown in the last column. For comparison, the last row shows the errors in total energy ΔE from the point nucleus value shown in the last column.

Energy	$a = 1.0$	$a = 2.0$	$a = 3.0$	$a = 5.0$	$a = 7.0$	point nucleus
$1s$	−0.3702	−0.0226	−0.0032	−0.0002	−0.0000	−113.8001
$2s$	0.0410	−0.0003	−0.0002	−0.0000	−0.0000	−10.7940
$2p$	0.0422	0.0024	0.0004	0.0000	0.0000	−8.4433
$3s$	0.0032	−0.0001	−0.0000	−0.0000	−0.0000	−0.8832
$3p$	0.0004	0.0001	0.0000	0.0000	0.0000	−0.3822
ΔE	0.7709388	0.0366550	0.0060785	0.0006401	0.0001421	−525.9397934

Table S36: Errors in positions of orbital density maxima in bohr for the Ar atom computed with PW92 and the regularized potential with various values of a. The values obtained with the Coulomb potential of the point nucleus are shown in the last column.

Energy	$a = 1.0$	$a = 2.0$	$a = 3.0$	$a = 5.0$	$a = 7.0$	point nucleus
$1s$	0.000130	−0.000436	−0.000005	−0.000000	0.000000	0.056677
$2s$	0.001455	0.000026	0.000002	0.000000	0.000000	0.341584
$2p$	0.002100	0.000112	0.000018	0.000002	0.000000	0.281415
$3s$	0.003625	0.000030	−0.000004	−0.000001	0.000000	1.178438
$3p$	0.003447	0.000234	0.000039	0.000003	0.000001	1.288244

Table S37: Errors in orbital energies in E_h for the Ar atom computed with PBE and the regularized potential with various values of a. The values obtained with the Coulomb potential of the point nucleus are shown in the last column. For comparison, the last row shows the errors in total energy ΔE from the point nucleus value shown in the last column.

Energy	$a = 1.0$	$a = 2.0$	$a = 3.0$	$a = 5.0$	$a = 7.0$	point nucleus
$1s$	−0.3587	−0.0198	−0.0021	0.0001	0.0001	−114.1646
$2s$	0.0429	−0.0001	−0.0001	0.0000	0.0000	−10.8310
$2p$	0.0417	0.0023	0.0004	0.0000	0.0000	−8.4437
$3s$	0.0034	−0.0001	−0.0000	−0.0000	0.0000	−0.8842
$3p$	0.0003	0.0001	0.0000	0.0000	0.0000	−0.3780
ΔE	0.8258155	0.0481923	0.0102538	0.0017119	0.0005641	−527.3461288

Table S38: Errors in positions of orbital density maxima in bohr for the Ar atom computed with PBE and the regularized potential with various values of a. The values obtained with the Coulomb potential of the point nucleus are shown in the last column.

Energy	$a = 1.0$	$a = 2.0$	$a = 3.0$	$a = 5.0$	$a = 7.0$	point nucleus
$1s$	0.000169	−0.000440	−0.000005	0.000000	0.000000	0.056535
$2s$	0.001493	0.000032	0.000003	0.000001	0.000000	0.340626
$2p$	0.002093	0.000111	0.000017	0.000002	0.000000	0.281656
$3s$	0.003753	0.000051	0.000002	0.000000	0.000000	1.181186
$3p$	0.003433	0.000227	0.000037	0.000003	0.000000	1.292594

Table S39: Errors in orbital energies in E_h for the Ar atom computed with TASKCC and the regularized potential with various values of a. The values obtained with the Coulomb potential of the point nucleus are shown in the last column. For comparison, the last row shows the errors in total energy ΔE from the point nucleus value shown in the last column.

Energy	$a = 1.0$	$a = 2.0$	$a = 3.0$	$a = 5.0$	$a = 7.0$	point nucleus
$1s$	−0.3474	−0.0211	−0.0029	−0.0002	−0.0000	−114.7941
$2s$	0.0432	−0.0002	−0.0002	−0.0000	−0.0000	−11.0871
$2p$	0.0429	0.0025	0.0004	0.0000	0.0000	−8.6358
$3s$	0.0035	−0.0001	−0.0000	−0.0000	−0.0000	−0.9492
$3p$	0.0004	0.0001	0.0000	0.0000	0.0000	−0.4104
ΔE	0.8067793	0.0400977	0.0066984	0.0006937	0.0001493	−527.3566109

Table S40: Errors in positions of orbital density maxima in bohr for the Ar atom computed with TASKCC and the regularized potential with various values of a. The values obtained with the Coulomb potential of the point nucleus are shown in the last column.

Energy	$a = 1.0$	$a = 2.0$	$a = 3.0$	$a = 5.0$	$a = 7.0$	point nucleus
$1s$	0.000167	−0.000439	−0.000005	−0.000000	0.000000	0.056557
$2s$	0.001489	0.000030	0.000002	0.000000	0.000000	0.340711
$2p$	0.002103	0.000113	0.000018	0.000001	0.000000	0.281575
$3s$	0.003778	0.000053	0.000000	0.000000	0.000000	1.189354
$3p$	0.003506	0.000233	0.000038	0.000003	0.000001	1.300650

Table S41: Errors in orbital energies in E_h for the Ca atom computed with HF and the regularized potential with various values of a. The values obtained with the Coulomb potential of the point nucleus are shown in the last column. For comparison, the last row shows the errors in total energy ΔE from the point nucleus value shown in the last column.

Energy	$a = 1.0$	$a = 2.0$	$a = 3.0$	$a = 5.0$	$a = 7.0$	point nucleus
$1s$	−0.3752	−0.0249	−0.0040	−0.0003	−0.0001	−149.3637
$2s$	0.0727	0.0001	−0.0002	−0.0000	−0.0000	−16.8227
$2p$	0.0681	0.0039	0.0006	0.0001	0.0000	−13.6293
$3s$	0.0084	−0.0000	−0.0000	−0.0000	−0.0000	−2.2454
$3p$	0.0023	0.0003	0.0001	0.0000	0.0000	−1.3407
$4s$	0.0004	−0.0000	−0.0000	−0.0000	−0.0000	−0.1955
ΔE	1.0751494	0.0464898	0.0067317	0.0005870	0.0001178	−676.7581859

Table S42: Errors in positions of orbital density maxima in bohr for the Ca atom computed with HF and the regularized potential with various values of a. The values obtained with the Coulomb potential of the point nucleus are shown in the last column.

Energy	$a = 1.0$	$a = 2.0$	$a = 3.0$	$a = 5.0$	$a = 7.0$	point nucleus
$1s$	0.000168	−0.000395	−0.000005	−0.000000	−0.000000	0.050834
$2s$	0.001406	0.000029	0.000002	0.000000	0.000000	0.300791
$2p$	0.001856	0.000099	0.000016	0.000001	0.000000	0.247673
$3s$	0.003418	0.000057	0.000001	−0.000001	−0.000000	0.996297
$3p$	0.002991	0.000193	0.000032	0.000003	0.000001	1.052815
$4s$	0.007769	−0.000005	−0.000027	−0.000004	−0.000001	3.475278

Table S43: Errors in orbital energies in E_h for the Ca atom computed with PW92 and the regularized potential with various values of a. The values obtained with the Coulomb potential of the point nucleus are shown in the last column. For comparison, the last row shows the errors in total energy ΔE from the point nucleus value shown in the last column.

Energy	$a = 1.0$	$a = 2.0$	$a = 3.0$	$a = 5.0$	$a = 7.0$	point nucleus
$1s$	−0.4281	−0.0264	−0.0038	−0.0002	−0.0000	−143.9351
$2s$	0.0626	0.0001	−0.0002	−0.0000	−0.0000	−15.0467
$2p$	0.0617	0.0035	0.0006	0.0001	0.0000	−12.2851
$3s$	0.0065	−0.0001	−0.0000	−0.0000	−0.0000	−1.7061
$3p$	0.0016	0.0002	0.0000	0.0000	0.0000	−1.0304
$4s$	0.0002	−0.0000	−0.0000	−0.0000	−0.0000	−0.1415
ΔE	1.0803757	0.0521100	0.0084837	0.0008620	0.0001874	−675.7353039

Table S44: Errors in positions of orbital density maxima in bohr for the Ca atom computed with PW92 and the regularized potential with various values of a. The values obtained with the Coulomb potential of the point nucleus are shown in the last column.

Energy	$a = 1.0$	$a = 2.0$	$a = 3.0$	$a = 5.0$	$a = 7.0$	point nucleus
$1s$	0.000142	−0.000394	−0.000005	−0.000000	0.000000	0.050924
$2s$	0.001367	0.000028	0.000002	0.000000	0.000000	0.302936
$2p$	0.001862	0.000100	0.000016	0.000001	0.000000	0.247380
$3s$	0.003356	0.000047	−0.000000	−0.000001	−0.000000	0.998581
$3p$	0.002948	0.000196	0.000033	0.000003	0.000001	1.047653
$4s$	0.007202	−0.000043	−0.000031	−0.000004	−0.000001	3.340236

Table S45: Errors in orbital energies in E_h for the Ca atom computed with PBE and the regularized potential with various values of a. The values obtained with the Coulomb potential of the point nucleus are shown in the last column. For comparison, the last row shows the errors in total energy ΔE from the point nucleus value shown in the last column.

Energy	$a = 1.0$	$a = 2.0$	$a = 3.0$	$a = 5.0$	$a = 7.0$	point nucleus
$1s$	−0.4147	−0.0232	−0.0025	0.0001	0.0001	−144.3495
$2s$	0.0650	0.0004	−0.0001	0.0000	0.0000	−15.0918
$2p$	0.0611	0.0035	0.0006	0.0000	0.0000	−12.2910
$3s$	0.0068	−0.0000	−0.0000	−0.0000	0.0000	−1.7157
$3p$	0.0015	0.0002	0.0000	0.0000	0.0000	−1.0311
$4s$	0.0002	0.0000	−0.0000	−0.0000	0.0000	−0.1379
ΔE	1.1431408	0.0652306	0.0132221	0.0020768	0.0006656	−677.3488191

Table S46: Errors in positions of orbital density maxima in bohr for the Ca atom computed with PBE and the regularized potential with various values of a. The values obtained with the Coulomb potential of the point nucleus are shown in the last column.

Energy	$a = 1.0$	$a = 2.0$	$a = 3.0$	$a = 5.0$	$a = 7.0$	point nucleus
$1s$	0.000174	−0.000397	−0.000004	0.000000	0.000000	0.050808
$2s$	0.001397	0.000032	0.000004	0.000000	0.000000	0.302133
$2p$	0.001857	0.000099	0.000016	0.000001	0.000000	0.247512
$3s$	0.003440	0.000062	0.000005	0.000001	0.000000	0.999356
$3p$	0.002942	0.000192	0.000031	0.000002	0.000000	1.049867
$4s$	0.007684	0.000001	−0.000018	−0.000001	0.000000	3.413933

Table S47: Errors in orbital energies in E_h for the Ca atom computed with TASKCC and the regularized potential with various values of a. The values obtained with the Coulomb potential of the point nucleus are shown in the last column. For comparison, the last row shows the errors in total energy ΔE from the point nucleus value shown in the last column.

Energy	$a = 1.0$	$a = 2.0$	$a = 3.0$	$a = 5.0$	$a = 7.0$	point nucleus
1s	−0.4009	−0.0246	−0.0034	−0.0002	−0.0000	−145.0976
2s	0.0655	0.0002	−0.0001	−0.0000	−0.0000	−15.4188
2p	0.0626	0.0036	0.0006	0.0001	0.0000	−12.5564
3s	0.0070	−0.0000	−0.0000	−0.0000	−0.0000	−1.8348
3p	0.0016	0.0002	0.0000	0.0000	0.0000	−1.1091
4s	0.0003	0.0000	−0.0000	−0.0000	−0.0000	−0.1454
ΔE	1.1216528	0.0561347	0.0092053	0.0009240	0.0001958	−677.2532220

Table S48: Errors in positions of orbital density maxima in bohr for the Ca atom computed with TASKCC and the regularized potential with various values of a. The values obtained with the Coulomb potential of the point nucleus are shown in the last column.

Energy	$a = 1.0$	$a = 2.0$	$a = 3.0$	$a = 5.0$	$a = 7.0$	point nucleus
1s	0.000171	−0.000396	−0.000004	−0.000000	0.000000	0.050830
2s	0.001395	0.000031	0.000003	0.000000	0.000000	0.302211
2p	0.001862	0.000101	0.000016	0.000001	0.000000	0.247389
3s	0.003451	0.000062	0.000003	0.000000	0.000000	1.003105
3p	0.002987	0.000197	0.000033	0.000003	0.000001	1.053457
4s	0.009015	−0.000049	−0.000038	−0.000005	−0.000001	3.669521

Table S49: Errors in orbital energies in E_h for the Zn atom computed with HF and the regularized potential with various values of a. The values obtained with the Coulomb potential of the point nucleus are shown in the last column. For comparison, the last row shows the errors in total energy ΔE from the point nucleus value shown in the last column.

Energy	$a = 1.0$	$a = 2.0$	$a = 3.0$	$a = 5.0$	$a = 7.0$	point nucleus
$1s$	−0.5958	−0.0409	−0.0066	−0.0005	−0.0001	−353.3045
$2s$	0.3070	0.0065	0.0004	−0.0000	−0.0000	−44.3617
$2p$	0.2712	0.0155	0.0025	0.0002	0.0000	−38.9248
$3s$	0.0590	0.0016	0.0001	0.0000	0.0000	−5.6378
$3p$	0.0319	0.0023	0.0004	0.0000	0.0000	−3.8394
$3d$	−0.0107	−0.0005	−0.0001	−0.0000	−0.0000	−0.7825
$4s$	0.0023	0.0001	0.0000	0.0000	0.0000	−0.2925
ΔE	3.2644482	0.1556870	0.0231720	0.0020104	0.0003960	−1777.8481162

Table S50: Errors in positions of orbital density maxima in bohr for the Zn atom computed with HF and the regularized potential with various values of a. The values obtained with the Coulomb potential of the point nucleus are shown in the last column.

Energy	$a = 1.0$	$a = 2.0$	$a = 3.0$	$a = 5.0$	$a = 7.0$	point nucleus
$1s$	0.000161	−0.000266	−0.000003	−0.000000	−0.000000	0.033722
$2s$	0.001056	0.000028	0.000002	0.000000	0.000000	0.191985
$2p$	0.001191	0.000066	0.000010	0.000001	0.000000	0.153583
$3s$	0.003081	0.000086	0.000008	0.000000	0.000000	0.596687
$3p$	0.002474	0.000161	0.000027	0.000003	0.000000	0.598608
$3d$	−0.001338	−0.000078	−0.000012	−0.000001	−0.000000	0.573959
$4s$	0.016189	0.000505	0.000052	0.000002	0.000000	2.267465

Table S51: Errors in orbital energies in E_h for the Zn atom computed with PW92 and the regularized potential with various values of a. The values obtained with the Coulomb potential of the point nucleus are shown in the last column. For comparison, the last row shows the errors in total energy ΔE from the point nucleus value shown in the last column.

Energy	$a = 1.0$	$a = 2.0$	$a = 3.0$	$a = 5.0$	$a = 7.0$	point nucleus
$1s$	−0.6860	−0.0440	−0.0064	−0.0004	−0.0001	−344.9698
$2s$	0.2881	0.0063	0.0005	0.0000	−0.0000	−41.5313
$2p$	0.2619	0.0150	0.0024	0.0002	0.0000	−36.6487
$3s$	0.0545	0.0015	0.0001	0.0000	0.0000	−4.5729
$3p$	0.0305	0.0022	0.0004	0.0000	0.0000	−3.0222
$3d$	−0.0074	−0.0003	−0.0000	−0.0000	−0.0000	−0.3988
$4s$	0.0022	0.0001	0.0000	0.0000	0.0000	−0.2227
ΔE	3.2655204	0.1640487	0.0258466	0.0024352	0.0005039	−1776.5614778

Table S52: Errors in positions of orbital density maxima in bohr for the Zn atom computed with PW92 and the regularized potential with various values of a. The values obtained with the Coulomb potential of the point nucleus are shown in the last column.

Energy	$a = 1.0$	$a = 2.0$	$a = 3.0$	$a = 5.0$	$a = 7.0$	point nucleus
1s	0.000150	−0.000266	−0.000003	−0.000000	0.000000	0.033761
2s	0.001037	0.000027	0.000003	0.000000	0.000000	0.192868
2p	0.001188	0.000066	0.000010	0.000001	0.000000	0.153474
3s	0.003085	0.000083	0.000008	0.000000	0.000000	0.597388
3p	0.002480	0.000165	0.000027	0.000003	0.000001	0.595704
3d	−0.001335	−0.000080	−0.000012	−0.000001	−0.000000	0.562133
4s	0.014769	0.000463	0.000050	0.000003	0.000001	2.115892

Table S53: Errors in orbital energies in E_h for the Zn atom computed with PBE and the regularized potential with various values of a. The values obtained with the Coulomb potential of the point nucleus are shown in the last column. For comparison, the last row shows the errors in total energy ΔE from the point nucleus value shown in the last column.

Energy	$a = 1.0$	$a = 2.0$	$a = 3.0$	$a = 5.0$	$a = 7.0$	point nucleus
1s	−0.6625	−0.0385	−0.0043	0.0001	0.0002	−345.6328
2s	0.2928	0.0070	0.0007	0.0001	0.0000	−41.6065
2p	0.2613	0.0149	0.0024	0.0002	0.0000	−36.6697
3s	0.0554	0.0016	0.0002	0.0000	0.0000	−4.5946
3p	0.0304	0.0022	0.0004	0.0000	0.0000	−3.0271
3d	−0.0074	−0.0003	−0.0000	−0.0000	−0.0000	−0.3883
4s	0.0022	0.0001	0.0000	0.0000	0.0000	−0.2141
ΔE	3.3690056	0.1851807	0.0334234	0.0043699	0.0012647	−1779.1827967

Table S54: Errors in positions of orbital density maxima in bohr for the Zn atom computed with PBE and the regularized potential with various values of a. The values obtained with the Coulomb potential of the point nucleus are shown in the last column.

Energy	$a = 1.0$	$a = 2.0$	$a = 3.0$	$a = 5.0$	$a = 7.0$	point nucleus
1s	0.000164	−0.000267	−0.000003	0.000000	0.000000	0.033710
2s	0.001051	0.000029	0.000003	0.000000	0.000000	0.192522
2p	0.001188	0.000066	0.000010	0.000001	0.000000	0.153485
3s	0.003111	0.000089	0.000009	0.000001	0.000000	0.596809
3p	0.002473	0.000164	0.000027	0.000003	0.000000	0.595945
3d	−0.001295	−0.000078	−0.000012	−0.000001	−0.000000	0.564979
4s	0.015642	0.000517	0.000061	0.000005	0.000001	2.131212

Table S55: Errors in orbital energies in E_h for the Zn atom computed with TASKCC and the regularized potential with various values of a. The values obtained with the Coulomb potential of the point nucleus are shown in the last column. For comparison, the last row shows the errors in total energy ΔE from the point nucleus value shown in the last column.

Energy	$a = 1.0$	$a = 2.0$	$a = 3.0$	$a = 5.0$	$a = 7.0$	point nucleus
$1s$	−0.6377	−0.0407	−0.0057	−0.0003	−0.0000	−346.6925
$2s$	0.2933	0.0067	0.0005	0.0000	0.0000	−42.0686
$2p$	0.2627	0.0152	0.0024	0.0002	0.0000	−37.0495
$3s$	0.0555	0.0016	0.0002	0.0000	0.0000	−4.7832
$3p$	0.0302	0.0022	0.0004	0.0000	0.0000	−3.1623
$3d$	−0.0083	−0.0004	−0.0001	−0.0000	−0.0000	−0.4067
$4s$	0.0028	0.0001	0.0000	0.0000	0.0000	−0.2374
ΔE	3.3280591	0.1705826	0.0270148	0.0025340	0.0005172	−1778.7847235

Table S56: Errors in positions of orbital density maxima in bohr for the Zn atom computed with TASKCC and the regularized potential with various values of a. The values obtained with the Coulomb potential of the point nucleus are shown in the last column.

Energy	$a = 1.0$	$a = 2.0$	$a = 3.0$	$a = 5.0$	$a = 7.0$	point nucleus
$1s$	0.000162	−0.000267	−0.000003	−0.000000	0.000000	0.033723
$2s$	0.001052	0.000029	0.000003	0.000000	0.000000	0.192629
$2p$	0.001189	0.000067	0.000011	0.000001	0.000000	0.153492
$3s$	0.003118	0.000087	0.000008	0.000000	0.000000	0.598052
$3p$	0.002476	0.000165	0.000027	0.000003	0.000001	0.597291
$3d$	−0.001267	−0.000077	−0.000012	−0.000001	−0.000000	0.567963
$4s$	0.018627	0.000625	0.000072	0.000005	0.000001	2.157472

Table S57: Errors in orbital energies in E_h for the Kr atom computed with HF and the regularized potential with various values of a. The values obtained with the Coulomb potential of the point nucleus are shown in the last column. For comparison, the last row shows the errors in total energy ΔE from the point nucleus value shown in the last column.

Energy	$a = 1.0$	$a = 2.0$	$a = 3.0$	$a = 5.0$	$a = 7.0$	point nucleus
$1s$	−0.7542	−0.0523	−0.0084	−0.0007	−0.0001	−520.1655
$2s$	0.4881	0.0111	0.0007	−0.0000	−0.0000	−69.9031
$2p$	0.4215	0.0243	0.0039	0.0004	0.0001	−63.0098
$3s$	0.0923	0.0022	0.0001	−0.0000	−0.0000	−10.8495
$3p$	0.0448	0.0033	0.0006	0.0001	0.0000	−8.3315
$3d$	−0.0313	−0.0018	−0.0003	−0.0000	−0.0000	−3.8252
$4s$	0.0075	0.0001	−0.0000	−0.0000	−0.0000	−1.1529
$4p$	0.0005	0.0001	0.0000	0.0000	0.0000	−0.5242
ΔE	5.2478688	0.2572838	0.0385769	0.0033455	0.0006563	−2752.0549773

Table S58: Errors in positions of orbital density maxima in bohr for the Kr atom computed with HF and the regularized potential with various values of a. The values obtained with the Coulomb potential of the point nucleus are shown in the last column.

Energy	$a = 1.0$	$a = 2.0$	$a = 3.0$	$a = 5.0$	$a = 7.0$	point nucleus
$1s$	0.000149	−0.000223	−0.000003	−0.000000	0.000000	0.028053
$2s$	0.000912	0.000025	0.000002	0.000000	0.000000	0.157808
$2p$	0.000980	0.000055	0.000009	0.000001	0.000000	0.125184
$3s$	0.002485	0.000072	0.000007	0.000000	0.000000	0.475424
$3p$	0.001813	0.000122	0.000020	0.000002	0.000000	0.467508
$3d$	−0.000627	−0.000040	−0.000006	−0.000000	−0.000000	0.413214
$4s$	0.006377	0.000164	0.000012	0.000000	0.000000	1.376905
$4p$	0.004037	0.000304	0.000052	0.000005	0.000001	1.565641

Table S59: Errors in orbital energies in E_h for the Kr atom computed with PW92 and the regularized potential with various values of a. The values obtained with the Coulomb potential of the point nucleus are shown in the last column. For comparison, the last row shows the errors in total energy ΔE from the point nucleus value shown in the last column.

Energy	$a = 1.0$	$a = 2.0$	$a = 3.0$	$a = 5.0$	$a = 7.0$	point nucleus
$1s$	−0.8679	−0.0564	−0.0082	−0.0006	−0.0001	−509.9831
$2s$	0.4627	0.0108	0.0008	0.0000	−0.0000	−66.2859
$2p$	0.4095	0.0235	0.0038	0.0003	0.0001	−60.0173
$3s$	0.0835	0.0019	0.0001	−0.0000	−0.0000	−9.3150
$3p$	0.0403	0.0030	0.0005	0.0000	0.0000	−7.0865
$3d$	−0.0289	−0.0017	−0.0003	−0.0000	−0.0000	−3.0739
$4s$	0.0065	0.0000	−0.0000	−0.0000	−0.0000	−0.8205
$4p$	0.0002	0.0001	0.0000	0.0000	0.0000	−0.3463
ΔE	5.2403164	0.2670035	0.0417684	0.0038580	0.0007869	−2750.1333061

Table S60: Errors in positions of orbital density maxima in bohr for the Kr atom computed with PW92 and the regularized potential with various values of a. The values obtained with the Coulomb potential of the point nucleus are shown in the last column.

Energy	$a = 1.0$	$a = 2.0$	$a = 3.0$	$a = 5.0$	$a = 7.0$	point nucleus
$1s$	0.000141	−0.000222	−0.000003	−0.000000	0.000000	0.028081
$2s$	0.000898	0.000025	0.000002	0.000000	0.000000	0.158392
$2p$	0.000977	0.000055	0.000009	0.000001	0.000000	0.125108
$3s$	0.002518	0.000072	0.000007	0.000000	0.000000	0.477492
$3p$	0.001835	0.000125	0.000021	0.000002	0.000000	0.467632
$3d$	−0.000627	−0.000041	−0.000007	−0.000001	−0.000000	0.409450
$4s$	0.006505	0.000153	0.000011	0.000000	0.000000	1.359649
$4p$	0.003924	0.000318	0.000055	0.000006	0.000001	1.553438

Table S61: Errors in orbital energies in E_h for the Kr atom computed with PBE and the regularized potential with various values of a. The values obtained with the Coulomb potential of the point nucleus are shown in the last column. For comparison, the last row shows the errors in total energy ΔE from the point nucleus value shown in the last column.

Energy	$a = 1.0$	$a = 2.0$	$a = 3.0$	$a = 5.0$	$a = 7.0$	point nucleus
1s	−0.8382	−0.0496	−0.0057	0.0001	0.0002	−510.7930
2s	0.4689	0.0116	0.0011	0.0001	0.0000	−66.3746
2p	0.4090	0.0235	0.0038	0.0003	0.0001	−60.0432
3s	0.0849	0.0021	0.0002	0.0000	0.0000	−9.3468
3p	0.0404	0.0030	0.0005	0.0000	0.0000	−7.0965
3d	−0.0290	−0.0017	−0.0003	−0.0000	−0.0000	−3.0630
4s	0.0065	0.0000	−0.0000	−0.0000	−0.0000	−0.8147
4p	0.0001	0.0000	0.0000	0.0000	0.0000	−0.3411
ΔE	5.3681294	0.2929986	0.0510716	0.0062308	0.0017197	−2753.4161089

Table S62: Errors in positions of orbital density maxima in bohr for the Kr atom computed with PBE and the regularized potential with various values of a. The values obtained with the Coulomb potential of the point nucleus are shown in the last column.

Energy	$a = 1.0$	$a = 2.0$	$a = 3.0$	$a = 5.0$	$a = 7.0$	point nucleus
1s	0.000151	−0.000223	−0.000002	0.000000	0.000000	0.028045
2s	0.000908	0.000026	0.000003	0.000000	0.000000	0.158157
2p	0.000977	0.000055	0.000009	0.000001	0.000000	0.125104
3s	0.002532	0.000075	0.000008	0.000001	0.000000	0.476901
3p	0.001829	0.000125	0.000021	0.000002	0.000000	0.467532
3d	−0.000618	−0.000040	−0.000006	−0.000001	−0.000000	0.410773
4s	0.006655	0.000170	0.000015	0.000001	0.000000	1.366808
4p	0.003986	0.000315	0.000054	0.000005	0.000001	1.560857

Table S63: Errors in orbital energies in E_h for the Kr atom computed with TASKCC and the regularized potential with various values of a. The values obtained with the Coulomb potential of the point nucleus are shown in the last column. For comparison, the last row shows the errors in total energy ΔE from the point nucleus value shown in the last column.

Energy	$a = 1.0$	$a = 2.0$	$a = 3.0$	$a = 5.0$	$a = 7.0$	point nucleus
1s	−0.8068	−0.0522	−0.0074	−0.0005	−0.0001	−512.1085
2s	0.4693	0.0113	0.0009	0.0000	0.0000	−67.0180
2p	0.4100	0.0238	0.0038	0.0003	0.0001	−60.5880
3s	0.0860	0.0021	0.0001	0.0000	−0.0000	−9.6297
3p	0.0408	0.0031	0.0005	0.0000	0.0000	−7.3197
3d	−0.0299	−0.0017	−0.0003	−0.0000	−0.0000	−3.1594
4s	0.0069	0.0000	−0.0000	−0.0000	−0.0000	−0.8760
4p	0.0001	0.0001	0.0000	0.0000	0.0000	−0.3711
ΔE	5.3182805	0.2753149	0.0432544	0.0039834	0.0008039	−2753.0465769

Table S64: Errors in positions of orbital density maxima in bohr for the Kr atom computed with TASKCC and the regularized potential with various values of a. The values obtained with the Coulomb potential of the point nucleus are shown in the last column.

Energy	$a = 1.0$	$a = 2.0$	$a = 3.0$	$a = 5.0$	$a = 7.0$	point nucleus
$1s$	0.000149	−0.000223	−0.000003	0.000000	0.000000	0.028055
$2s$	0.000909	0.000026	0.000003	0.000000	0.000000	0.158252
$2p$	0.000977	0.000055	0.000009	0.000001	0.000000	0.125131
$3s$	0.002528	0.000074	0.000007	0.000000	0.000000	0.477387
$3p$	0.001820	0.000125	0.000021	0.000002	0.000000	0.467845
$3d$	−0.000611	−0.000040	−0.000006	−0.000001	−0.000000	0.411521
$4s$	0.006835	0.000184	0.000016	0.000001	0.000000	1.376068
$4p$	0.004248	0.000330	0.000057	0.000006	0.000001	1.562354

Table S65: Errors in orbital energies in E_h for the Sr atom computed with HF and the regularized potential with various values of a. The values obtained with the Coulomb potential of the point nucleus are shown in the last column. For comparison, the last row shows the errors in total energy ΔE from the point nucleus value shown in the last column.

Energy	$a = 1.0$	$a = 2.0$	$a = 3.0$	$a = 5.0$	$a = 7.0$	point nucleus
$1s$	−0.8079	−0.0561	−0.0090	−0.0008	−0.0001	−583.6879
$2s$	0.5597	0.0131	0.0009	−0.0000	−0.0000	−80.3908
$2p$	0.4806	0.0278	0.0045	0.0004	0.0001	−72.9960
$3s$	0.1081	0.0026	0.0002	−0.0000	−0.0000	−13.4750
$3p$	0.0517	0.0039	0.0007	0.0001	0.0000	−10.7000
$3d$	−0.0380	−0.0022	−0.0003	−0.0000	−0.0000	−5.6944
$4s$	0.0108	0.0002	−0.0000	−0.0000	−0.0000	−1.8968
$4p$	0.0010	0.0002	0.0000	0.0000	0.0000	−1.0982
$5s$	0.0006	0.0000	−0.0000	−0.0000	−0.0000	−0.1785
ΔE	6.0345652	0.2979414	0.0447537	0.0038809	0.0007606	−3131.5456864

Table S66: Errors in positions of orbital density maxima in bohr for the Sr atom computed with HF and the regularized potential with various values of a. The values obtained with the Coulomb potential of the point nucleus are shown in the last column.

Energy	$a = 1.0$	$a = 2.0$	$a = 3.0$	$a = 5.0$	$a = 7.0$	point nucleus
$1s$	0.000144	−0.000211	−0.000002	−0.000000	0.000000	0.026565
$2s$	0.000872	0.000025	0.000002	0.000000	0.000000	0.148959
$2p$	0.000924	0.000052	0.000008	0.000001	0.000000	0.117913
$3s$	0.002328	0.000069	0.000007	0.000000	0.000000	0.444452
$3p$	0.001659	0.000113	0.000019	0.000002	0.000000	0.434878
$3d$	−0.000506	−0.000034	−0.000005	−0.000001	−0.000000	0.378131
$4s$	0.005368	0.000143	0.000012	0.000000	0.000000	1.217484
$4p$	0.003311	0.000249	0.000042	0.000004	0.000000	1.337044
$5s$	0.012959	0.000174	−0.000010	−0.000004	−0.000001	3.862631

Table S67: Errors in orbital energies in E_h for the Sr atom computed with PW92 and the regularized potential with various values of a. The values obtained with the Coulomb potential of the point nucleus are shown in the last column. For comparison, the last row shows the errors in total energy ΔE from the point nucleus value shown in the last column.

Energy	$a = 1.0$	$a = 2.0$	$a = 3.0$	$a = 5.0$	$a = 7.0$	point nucleus
$1s$	−0.9295	−0.0606	−0.0088	−0.0006	−0.0001	−572.8702
$2s$	0.5320	0.0127	0.0010	0.0000	−0.0000	−76.4916
$2p$	0.4677	0.0270	0.0044	0.0004	0.0001	−69.7458
$3s$	0.0978	0.0023	0.0002	−0.0000	−0.0000	−11.7713
$3p$	0.0463	0.0036	0.0006	0.0001	0.0000	−9.3016
$3d$	−0.0359	−0.0020	−0.0003	−0.0000	−0.0000	−4.8132
$4s$	0.0094	0.0001	−0.0000	−0.0000	−0.0000	−1.4551
$4p$	0.0005	0.0002	0.0000	0.0000	0.0000	−0.8443
$5s$	0.0004	0.0000	−0.0000	−0.0000	−0.0000	−0.1319
ΔE	6.0243861	0.3081219	0.0481193	0.0044229	0.0008988	−3129.4380223

Table S68: Errors in positions of orbital density maxima in bohr for the Sr atom computed with PW92 and the regularized potential with various values of a. The values obtained with the Coulomb potential of the point nucleus are shown in the last column.

Energy	$a = 1.0$	$a = 2.0$	$a = 3.0$	$a = 5.0$	$a = 7.0$	point nucleus
$1s$	0.000137	−0.000211	−0.000002	−0.000000	0.000000	0.026589
$2s$	0.000859	0.000024	0.000002	0.000000	0.000000	0.149479
$2p$	0.000922	0.000052	0.000008	0.000001	0.000000	0.117848
$3s$	0.002359	0.000068	0.000007	0.000000	0.000000	0.446670
$3p$	0.001678	0.000115	0.000019	0.000002	0.000000	0.435391
$3d$	−0.000505	−0.000034	−0.000005	−0.000001	−0.000000	0.375268
$4s$	0.005517	0.000136	0.000010	0.000000	0.000000	1.211884
$4p$	0.003240	0.000257	0.000044	0.000004	0.000001	1.331663
$5s$	0.012516	0.000133	−0.000015	−0.000004	−0.000001	3.678856

Table S69: Errors in orbital energies in E_h for the Sr atom computed with PBE and the regularized potential with various values of a. The values obtained with the Coulomb potential of the point nucleus are shown in the last column. For comparison, the last row shows the errors in total energy ΔE from the point nucleus value shown in the last column.

Energy	$a = 1.0$	$a = 2.0$	$a = 3.0$	$a = 5.0$	$a = 7.0$	point nucleus
$1s$	−0.8978	−0.0533	−0.0061	0.0001	0.0002	−573.7319
$2s$	0.5387	0.0136	0.0013	0.0001	0.0000	−76.5884
$2p$	0.4672	0.0269	0.0043	0.0004	0.0001	−69.7769
$3s$	0.0993	0.0025	0.0002	0.0000	0.0000	−11.8088
$3p$	0.0464	0.0036	0.0006	0.0001	0.0000	−9.3161
$3d$	−0.0361	−0.0021	−0.0003	−0.0000	−0.0000	−4.8056
$4s$	0.0094	0.0001	−0.0000	−0.0000	−0.0000	−1.4552
$4p$	0.0004	0.0002	0.0000	0.0000	0.0000	−0.8431
$5s$	0.0003	0.0000	−0.0000	−0.0000	−0.0000	−0.1278
ΔE	6.1604440	0.3357622	0.0580049	0.0069431	0.0018894	−3132.9486592

Table S70: Errors in positions of orbital density maxima in bohr for the Sr atom computed with PBE and the regularized potential with various values of a. The values obtained with the Coulomb potential of the point nucleus are shown in the last column.

Energy	$a = 1.0$	$a = 2.0$	$a = 3.0$	$a = 5.0$	$a = 7.0$	point nucleus
$1s$	0.000146	−0.000212	−0.000002	0.000000	0.000000	0.026557
$2s$	0.000867	0.000026	0.000003	0.000000	0.000000	0.149269
$2p$	0.000922	0.000052	0.000008	0.000001	0.000000	0.117842
$3s$	0.002371	0.000072	0.000008	0.000001	0.000000	0.446088
$3p$	0.001673	0.000115	0.000019	0.000002	0.000000	0.435234
$3d$	−0.000498	−0.000033	−0.000005	−0.000000	−0.000000	0.376330
$4s$	0.005596	0.000148	0.000014	0.000001	0.000001	1.216066
$4p$	0.003281	0.000255	0.000044	0.000004	0.000001	1.335481
$5s$	0.013269	0.000162	−0.000008	−0.000002	0.000000	3.766503

Table S71: Errors in orbital energies in E_h for the Sr atom computed with TASKCC and the regularized potential with various values of a. The values obtained with the Coulomb potential of the point nucleus are shown in the last column. For comparison, the last row shows the errors in total energy ΔE from the point nucleus value shown in the last column.

Energy	$a = 1.0$	$a = 2.0$	$a = 3.0$	$a = 5.0$	$a = 7.0$	point nucleus
$1s$	−0.8641	−0.0560	−0.0080	−0.0005	−0.0001	−575.1713
$2s$	0.5392	0.0132	0.0011	0.0000	0.0000	−77.3286
$2p$	0.4682	0.0273	0.0044	0.0004	0.0001	−70.4141
$3s$	0.1008	0.0025	0.0002	0.0000	−0.0000	−12.1573
$3p$	0.0470	0.0036	0.0006	0.0001	0.0000	−9.6038
$3d$	−0.0369	−0.0021	−0.0003	−0.0000	−0.0000	−4.9646
$4s$	0.0099	0.0001	−0.0000	−0.0000	−0.0000	−1.5654
$4p$	0.0004	0.0002	0.0000	0.0000	0.0000	−0.9153
$5s$	0.0004	0.0000	−0.0000	−0.0000	−0.0000	−0.1337
ΔE	6.1073567	0.3170417	0.0497152	0.0045576	0.0009172	−3132.4842944

Table S72: Errors in positions of orbital density maxima in bohr for the Sr atom computed with TASKCC and the regularized potential with various values of a. The values obtained with the Coulomb potential of the point nucleus are shown in the last column.

Energy	$a = 1.0$	$a = 2.0$	$a = 3.0$	$a = 5.0$	$a = 7.0$	point nucleus
$1s$	0.000144	−0.000211	−0.000002	0.000000	0.000000	0.026567
$2s$	0.000868	0.000025	0.000003	0.000000	0.000000	0.149357
$2p$	0.000922	0.000053	0.000008	0.000001	0.000000	0.117869
$3s$	0.002367	0.000070	0.000007	0.000000	0.000000	0.446475
$3p$	0.001665	0.000115	0.000019	0.000002	0.000000	0.435444
$3d$	−0.000494	−0.000033	−0.000005	−0.000001	−0.000000	0.376866
$4s$	0.005672	0.000155	0.000014	0.000001	0.000000	1.220887
$4p$	0.003421	0.000263	0.000045	0.000004	0.000001	1.335444
$5s$	0.016730	0.000139	−0.000029	−0.000006	−0.000002	4.065596

Table S73: Errors in orbital energies in E_h for the Cd atom computed with HF and the regularized potential with various values of a. The values obtained with the Coulomb potential of the point nucleus are shown in the last column. For comparison, the last row shows the errors in total energy ΔE from the point nucleus value shown in the last column.

Energy	$a = 1.0$	$a = 2.0$	$a = 3.0$	$a = 5.0$	$a = 7.0$	point nucleus
$1s$	−1.0584	−0.0744	−0.0120	−0.0010	−0.0002	−955.3154
$2s$	1.0246	0.0270	0.0023	0.0001	−0.0000	−142.0068
$2p$	0.8653	0.0507	0.0082	0.0007	0.0001	−132.0470
$3s$	0.2370	0.0067	0.0006	0.0000	−0.0000	−27.7086
$3p$	0.1209	0.0091	0.0016	0.0001	0.0000	−23.5972
$3d$	−0.0523	−0.0032	−0.0005	−0.0000	−0.0000	−16.0720
$4s$	0.0449	0.0014	0.0001	0.0000	0.0000	−4.4505
$4p$	0.0168	0.0015	0.0003	0.0000	0.0000	−3.0535
$4d$	−0.0082	−0.0004	−0.0001	−0.0000	−0.0000	−0.7637
$5s$	0.0024	0.0001	0.0000	0.0000	0.0000	−0.2649
ΔE	10.7672549	0.5446860	0.0823369	0.0071407	0.0013954	−5465.1331425

Table S74: Errors in positions of orbital density maxima in bohr for the Cd atom computed with HF and the regularized potential with various values of a. The values obtained with the Coulomb potential of the point nucleus are shown in the last column.

Energy	$a = 1.0$	$a = 2.0$	$a = 3.0$	$a = 5.0$	$a = 7.0$	point nucleus
$1s$	0.000125	−0.000168	−0.000002	−0.000000	0.000000	0.020993
$2s$	0.000712	0.000021	0.000002	0.000000	0.000000	0.116292
$2p$	0.000720	0.000041	0.000007	0.000001	0.000000	0.091332
$3s$	0.001788	0.000055	0.000006	0.000000	0.000000	0.334770
$3p$	0.001177	0.000082	0.000014	0.000001	0.000000	0.322031
$3d$	−0.000199	−0.000016	−0.000003	−0.000000	0.000000	0.266427
$4s$	0.003935	0.000121	0.000012	0.000000	0.000000	0.822684
$4p$	0.002421	0.000181	0.000031	0.000003	0.000001	0.859507
$4d$	−0.001573	−0.000101	−0.000016	−0.000001	−0.000000	0.977260
$5s$	0.019640	0.000649	0.000069	0.000004	0.000001	2.583744

Table S75: Errors in orbital energies in E_h for the Cd atom computed with PW92 and the regularized potential with various values of a. The values obtained with the Coulomb potential of the point nucleus are shown in the last column. For comparison, the last row shows the errors in total energy ΔE from the point nucleus value shown in the last column.

Energy	$a = 1.0$	$a = 2.0$	$a = 3.0$	$a = 5.0$	$a = 7.0$	point nucleus
$1s$	−1.2187	−0.0806	−0.0118	−0.0008	−0.0001	−941.4768
$2s$	0.9873	0.0265	0.0024	0.0001	0.0000	−136.8325
$2p$	0.8498	0.0497	0.0080	0.0007	0.0001	−127.6351
$3s$	0.2206	0.0063	0.0006	0.0000	0.0000	−25.3798
$3p$	0.1125	0.0085	0.0015	0.0001	0.0000	−21.6374
$3d$	−0.0498	−0.0030	−0.0005	−0.0000	−0.0000	−14.6851
$4s$	0.0434	0.0013	0.0001	0.0000	0.0000	−3.5959
$4p$	0.0171	0.0015	0.0003	0.0000	0.0000	−2.3951
$4d$	−0.0062	−0.0003	−0.0000	−0.0000	−0.0000	−0.4704
$5s$	0.0022	0.0001	0.0000	0.0000	0.0000	−0.2042
ΔE	10.7482876	0.5574430	0.0866174	0.0078343	0.0015726	−5462.3711357

Table S76: Errors in positions of orbital density maxima in bohr for the Cd atom computed with PW92 and the regularized potential with various values of a. The values obtained with the Coulomb potential of the point nucleus are shown in the last column.

Energy	$a = 1.0$	$a = 2.0$	$a = 3.0$	$a = 5.0$	$a = 7.0$	point nucleus
$1s$	0.000121	−0.000168	−0.000002	−0.000000	0.000000	0.021009
$2s$	0.000703	0.000021	0.000002	0.000000	0.000000	0.116595
$2p$	0.000718	0.000041	0.000007	0.000001	0.000000	0.091287
$3s$	0.001800	0.000055	0.000006	0.000000	0.000000	0.336464
$3p$	0.001180	0.000083	0.000014	0.000001	0.000000	0.322771
$3d$	−0.000195	−0.000016	−0.000003	−0.000000	−0.000000	0.265871
$4s$	0.004024	0.000119	0.000011	0.000000	0.000000	0.821070
$4p$	0.002439	0.000188	0.000032	0.000003	0.000001	0.855640
$4d$	−0.001837	−0.000119	−0.000019	−0.000002	−0.000000	0.974533
$5s$	0.016894	0.000554	0.000060	0.000004	0.000001	2.426584

Table S77: Errors in orbital energies in E_h for the Cd atom computed with PBE and the regularized potential with various values of a. The values obtained with the Coulomb potential of the point nucleus are shown in the last column. For comparison, the last row shows the errors in total energy ΔE from the point nucleus value shown in the last column.

Energy	$a = 1.0$	$a = 2.0$	$a = 3.0$	$a = 5.0$	$a = 7.0$	point nucleus
1s	−1.1768	−0.0711	−0.0083	0.0001	0.0003	−942.5807
2s	0.9963	0.0277	0.0028	0.0002	0.0000	−136.9562
2p	0.8491	0.0496	0.0080	0.0007	0.0001	−127.6759
3s	0.2232	0.0066	0.0007	0.0000	0.0000	−25.4181
3p	0.1127	0.0085	0.0015	0.0001	0.0000	−21.6485
3d	−0.0502	−0.0030	−0.0005	−0.0000	−0.0000	−14.6734
4s	0.0433	0.0014	0.0001	0.0000	0.0000	−3.6013
4p	0.0169	0.0015	0.0003	0.0000	0.0000	−2.3951
4d	−0.0062	−0.0003	−0.0000	−0.0000	−0.0000	−0.4645
5s	0.0021	0.0001	0.0000	0.0000	0.0000	−0.1940
ΔE	10.9260825	0.5933252	0.0994183	0.0110927	0.0028529	−5467.0515829

Table S78: Errors in positions of orbital density maxima in bohr for the Cd atom computed with PBE and the regularized potential with various values of a. The values obtained with the Coulomb potential of the point nucleus are shown in the last column.

Energy	$a = 1.0$	$a = 2.0$	$a = 3.0$	$a = 5.0$	$a = 7.0$	point nucleus
1s	0.000126	−0.000168	−0.000002	0.000000	0.000000	0.020988
2s	0.000709	0.000022	0.000002	0.000000	0.000000	0.116470
2p	0.000719	0.000041	0.000007	0.000001	0.000000	0.091278
3s	0.001805	0.000057	0.000006	0.000000	0.000000	0.336003
3p	0.001177	0.000083	0.000014	0.000001	0.000000	0.322543
3d	−0.000195	−0.000016	−0.000003	−0.000000	0.000000	0.266251
4s	0.004027	0.000123	0.000013	0.000001	0.000000	0.821914
4p	0.002432	0.000186	0.000032	0.000003	0.000001	0.856916
4d	−0.001741	−0.000114	−0.000018	−0.000002	−0.000000	0.975060
5s	0.017794	0.000603	0.000069	0.000005	0.000001	2.443583

Table S79: Errors in orbital energies in E_h for the Cd atom computed with TASKCC and the regularized potential with various values of a. The values obtained with the Coulomb potential of the point nucleus are shown in the last column. For comparison, the last row shows the errors in total energy ΔE from the point nucleus value shown in the last column.

Energy	$a = 1.0$	$a = 2.0$	$a = 3.0$	$a = 5.0$	$a = 7.0$	point nucleus
1s	−1.1327	−0.0743	−0.0107	−0.0007	−0.0001	−944.3845
2s	0.9966	0.0272	0.0025	0.0001	0.0000	−137.9301
2p	0.8492	0.0500	0.0081	0.0007	0.0001	−128.5216
3s	0.2253	0.0066	0.0006	0.0000	0.0000	−25.8327
3p	0.1133	0.0087	0.0015	0.0001	0.0000	−21.9992
3d	−0.0516	−0.0031	−0.0005	−0.0000	−0.0000	−14.8893
4s	0.0430	0.0013	0.0001	0.0000	0.0000	−3.7500
4p	0.0164	0.0014	0.0003	0.0000	0.0000	−2.5068
4d	−0.0069	−0.0003	−0.0000	−0.0000	−0.0000	−0.4941
5s	0.0026	0.0001	0.0000	0.0000	0.0000	−0.2116
ΔE	10.8525107	0.5690307	0.0886970	0.0080088	0.0015962	−5466.3121236

Table S80: Errors in positions of orbital density maxima in bohr for the Cd atom computed with TASKCC and the regularized potential with various values of a. The values obtained with the Coulomb potential of the point nucleus are shown in the last column.

Energy	$a = 1.0$	$a = 2.0$	$a = 3.0$	$a = 5.0$	$a = 7.0$	point nucleus
1s	0.000125	−0.000168	−0.000002	0.000000	0.000000	0.020995
2s	0.000709	0.000022	0.000002	0.000000	0.000000	0.116536
2p	0.000718	0.000041	0.000007	0.000001	0.000000	0.091305
3s	0.001801	0.000056	0.000006	0.000000	0.000000	0.336151
3p	0.001172	0.000083	0.000014	0.000001	0.000000	0.322573
3d	−0.000194	−0.000016	−0.000003	−0.000000	−0.000000	0.266346
4s	0.004011	0.000122	0.000012	0.000001	0.000000	0.824853
4p	0.002430	0.000186	0.000032	0.000003	0.000001	0.859683
4d	−0.001637	−0.000108	−0.000017	−0.000001	−0.000000	0.975390
5s	0.022381	0.000765	0.000086	0.000005	0.000001	2.449810

Table S81: Errors in orbital energies in E_h for the Xe atom computed with HF and the regularized potential with various values of a. The values obtained with the Coulomb potential of the point nucleus are shown in the last column. For comparison, the last row shows the errors in total energy ΔE from the point nucleus value shown in the last column.

Energy	$a = 1.0$	$a = 2.0$	$a = 3.0$	$a = 5.0$	$a = 7.0$	point nucleus
$1s$	−1.2223	−0.0864	−0.0140	−0.0012	−0.0002	−1224.3978
$2s$	1.3612	0.0369	0.0033	0.0001	0.0000	−189.3401
$2p$	1.1398	0.0670	0.0108	0.0010	0.0002	−177.7824
$3s$	0.3273	0.0093	0.0008	0.0000	−0.0000	−40.1757
$3p$	0.1630	0.0124	0.0021	0.0002	0.0000	−35.2217
$3d$	−0.0715	−0.0046	−0.0007	−0.0001	−0.0000	−26.1189
$4s$	0.0667	0.0018	0.0001	0.0000	−0.0000	−7.8563
$4p$	0.0229	0.0020	0.0004	0.0000	0.0000	−6.0083
$4d$	−0.0189	−0.0012	−0.0002	−0.0000	−0.0000	−2.7779
$5s$	0.0071	0.0001	−0.0000	−0.0000	−0.0000	−0.9444
$5p$	0.0003	0.0001	0.0000	0.0000	0.0000	−0.4573
ΔE	14.3548617	0.7337275	0.1112116	0.0096472	0.0018832	−7232.1383639

Table S82: Errors in positions of orbital density maxima in bohr for the Xe atom computed with HF and the regularized potential with various values of a. The values obtained with the Coulomb potential of the point nucleus are shown in the last column.

Energy	$a = 1.0$	$a = 2.0$	$a = 3.0$	$a = 5.0$	$a = 7.0$	point nucleus
$1s$	0.000115	−0.000149	−0.000002	−0.000000	0.000000	0.018646
$2s$	0.000640	0.000019	0.000002	0.000000	0.000000	0.102751
$2p$	0.000635	0.000037	0.000006	0.000001	0.000000	0.080437
$3s$	0.001573	0.000050	0.000005	0.000000	0.000000	0.291303
$3p$	0.001002	0.000071	0.000012	0.000001	0.000000	0.278426
$3d$	−0.000121	−0.000011	−0.000002	−0.000000	−0.000000	0.226670
$4s$	0.003203	0.000101	0.000010	0.000000	0.000000	0.687112
$4p$	0.001838	0.000140	0.000024	0.000002	0.000000	0.704892
$4d$	−0.000809	−0.000056	−0.000009	−0.000001	−0.000000	0.746845
$5s$	0.008100	0.000233	0.000020	0.000000	0.000000	1.713121
$5p$	0.004144	0.000349	0.000061	0.000006	0.000002	1.939680

Table S83: Errors in orbital energies in E_h for the Xe atom computed with PW92 and the regularized potential with various values of a. The values obtained with the Coulomb potential of the point nucleus are shown in the last column. For comparison, the last row shows the errors in total energy ΔE from the point nucleus value shown in the last column.

Energy	$a = 1.0$	$a = 2.0$	$a = 3.0$	$a = 5.0$	$a = 7.0$	point nucleus
1s	−1.4064	−0.0937	−0.0138	−0.0010	−0.0001	−1208.6891
2s	1.3173	0.0362	0.0034	0.0001	0.0000	−183.3275
2p	1.1219	0.0659	0.0106	0.0010	0.0002	−172.5996
3s	0.3068	0.0088	0.0008	0.0000	0.0000	−37.4153
3p	0.1525	0.0116	0.0020	0.0002	0.0000	−32.8669
3d	−0.0695	−0.0045	−0.0007	−0.0001	−0.0000	−24.3781
4s	0.0630	0.0016	0.0001	0.0000	−0.0000	−6.6782
4p	0.0218	0.0019	0.0003	0.0000	0.0000	−5.0636
4d	−0.0175	−0.0011	−0.0002	−0.0000	−0.0000	−2.2865
5s	0.0063	0.0000	−0.0000	−0.0000	−0.0000	−0.6720
5p	0.0000	0.0000	0.0000	0.0000	0.0000	−0.3098
ΔE	14.3255451	0.7477595	0.1160029	0.0104290	0.0020834	−7228.8341637

Table S84: Errors in positions of orbital density maxima in bohr for the Xe atom computed with PW92 and the regularized potential with various values of a. The values obtained with the Coulomb potential of the point nucleus are shown in the last column.

Energy	$a = 1.0$	$a = 2.0$	$a = 3.0$	$a = 5.0$	$a = 7.0$	point nucleus
1s	0.000112	−0.000149	−0.000002	−0.000000	0.000000	0.018658
2s	0.000633	0.000019	0.000002	0.000000	0.000000	0.102982
2p	0.000634	0.000037	0.000006	0.000001	0.000000	0.080402
3s	0.001578	0.000050	0.000005	0.000000	0.000000	0.292694
3p	0.001002	0.000071	0.000012	0.000001	0.000000	0.279112
3d	−0.000119	−0.000011	−0.000002	−0.000000	0.000000	0.226503
4s	0.003310	0.000101	0.000010	0.000000	0.000000	0.688477
4p	0.001874	0.000147	0.000025	0.000002	0.000001	0.705072
4d	−0.000911	−0.000063	−0.000010	−0.000001	−0.000000	0.745465
5s	0.008332	0.000219	0.000017	0.000000	0.000000	1.686624
5p	0.003849	0.000358	0.000064	0.000006	0.000001	1.924671

Table S85: Errors in orbital energies in E_h for the Xe atom computed with PBE and the regularized potential with various values of a. The values obtained with the Coulomb potential of the point nucleus are shown in the last column. For comparison, the last row shows the errors in total energy ΔE from the point nucleus value shown in the last column.

Energy	$a = 1.0$	$a = 2.0$	$a = 3.0$	$a = 5.0$	$a = 7.0$	point nucleus
$1s$	−1.3583	−0.0828	−0.0098	0.0001	0.0003	−1209.9445
$2s$	1.3279	0.0376	0.0038	0.0002	0.0001	−183.4753
$2p$	1.1214	0.0658	0.0106	0.0010	0.0002	−172.6537
$3s$	0.3101	0.0091	0.0009	0.0000	0.0000	−37.4570
$3p$	0.1528	0.0116	0.0020	0.0002	0.0000	−32.8802
$3d$	−0.0699	−0.0045	−0.0007	−0.0001	−0.0000	−24.3707
$4s$	0.0633	0.0017	0.0001	0.0000	0.0000	−6.6979
$4p$	0.0217	0.0019	0.0003	0.0000	0.0000	−5.0731
$4d$	−0.0175	−0.0011	−0.0002	−0.0000	−0.0000	−2.2840
$5s$	0.0062	0.0001	−0.0000	−0.0000	−0.0000	−0.6646
$5p$	−0.0000	0.0000	0.0000	0.0000	0.0000	−0.3046
ΔE	14.5286158	0.7886246	0.1305652	0.0141333	0.0035386	−7234.2332120

Table S86: Errors in positions of orbital density maxima in bohr for the Xe atom computed with PBE and the regularized potential with various values of a. The values obtained with the Coulomb potential of the point nucleus are shown in the last column.

Energy	$a = 1.0$	$a = 2.0$	$a = 3.0$	$a = 5.0$	$a = 7.0$	point nucleus
$1s$	0.000116	−0.000150	−0.000002	0.000000	0.000000	0.018643
$2s$	0.000638	0.000020	0.000002	0.000000	0.000000	0.102887
$2p$	0.000634	0.000037	0.000006	0.000001	0.000000	0.080394
$3s$	0.001582	0.000051	0.000006	0.000000	0.000000	0.292305
$3p$	0.001000	0.000071	0.000012	0.000001	0.000000	0.278896
$3d$	−0.000119	−0.000011	−0.000002	−0.000000	0.000000	0.226728
$4s$	0.003302	0.000104	0.000011	0.000001	0.000000	0.688525
$4p$	0.001864	0.000145	0.000025	0.000002	0.000000	0.705356
$4d$	−0.000868	−0.000061	−0.000010	−0.000001	−0.000000	0.745762
$5s$	0.008527	0.000236	0.000021	0.000001	0.000000	1.696320
$5p$	0.003968	0.000359	0.000063	0.000006	0.000001	1.934559

Table S87: Errors in orbital energies in E_h for the Xe atom computed with TASKCC and the regularized potential with various values of a. The values obtained with the Coulomb potential of the point nucleus are shown in the last column. For comparison, the last row shows the errors in total energy ΔE from the point nucleus value shown in the last column.

Energy	$a = 1.0$	$a = 2.0$	$a = 3.0$	$a = 5.0$	$a = 7.0$	point nucleus
$1s$	−1.3075	−0.0864	−0.0126	−0.0008	−0.0001	−1212.0214
$2s$	1.3285	0.0370	0.0035	0.0001	0.0000	−184.6493
$2p$	1.1213	0.0662	0.0107	0.0010	0.0002	−173.6861
$3s$	0.3128	0.0090	0.0008	0.0000	0.0000	−37.9716
$3p$	0.1536	0.0118	0.0020	0.0002	0.0000	−33.3301
$3d$	−0.0712	−0.0046	−0.0007	−0.0001	−0.0000	−24.6824
$4s$	0.0637	0.0017	0.0001	0.0000	−0.0000	−6.9179
$4p$	0.0217	0.0019	0.0003	0.0000	0.0000	−5.2477
$4d$	−0.0178	−0.0011	−0.0002	−0.0000	−0.0000	−2.3639
$5s$	0.0066	0.0001	−0.0000	−0.0000	−0.0000	−0.7190
$5p$	−0.0000	0.0000	0.0000	0.0000	0.0000	−0.3306
ΔE	14.4457645	0.7612456	0.1184265	0.0106325	0.0021111	−7233.3416395

Table S88: Errors in positions of orbital density maxima in bohr for the Xe atom computed with TASKCC and the regularized potential with various values of a. The values obtained with the Coulomb potential of the point nucleus are shown in the last column.

Energy	$a = 1.0$	$a = 2.0$	$a = 3.0$	$a = 5.0$	$a = 7.0$	point nucleus
$1s$	0.000115	−0.000150	−0.000002	−0.000000	0.000000	0.018648
$2s$	0.000638	0.000020	0.000002	0.000000	0.000000	0.102941
$2p$	0.000634	0.000037	0.000006	0.000001	0.000000	0.080418
$3s$	0.001579	0.000050	0.000005	0.000000	0.000000	0.292393
$3p$	0.000996	0.000071	0.000012	0.000001	0.000000	0.278905
$3d$	−0.000118	−0.000011	−0.000002	−0.000000	0.000000	0.226757
$4s$	0.003284	0.000102	0.000010	0.000000	0.000000	0.689832
$4p$	0.001849	0.000145	0.000025	0.000002	0.000001	0.706492
$4d$	−0.000837	−0.000059	−0.000009	−0.000001	−0.000000	0.746263
$5s$	0.008732	0.000256	0.000024	0.000001	0.000000	1.709528
$5p$	0.004292	0.000377	0.000067	0.000007	0.000002	1.937097
